# Single-atom nanozyme-mediated dihydroartemisinin delivery for self-enhanced chemodynamic therapy and ferroptosis

**DOI:** 10.1016/j.mtbio.2025.102096

**Published:** 2025-07-22

**Authors:** Yuanlong Zhang, Binghong Chen, Penghui Wei, Zhongyuan Shen, Xiyue Wu, Wenzhong Mei, Yang Zhu, Yuanxiang Lin

**Affiliations:** a Department of Neurosurgery, Neurosurgery Research Institute, The First Affiliated Hospital, Fujian Medical University, Fuzhou, 350005, China; b Department of Neurosurgery, National Regional Medical Center, Binhai Campus of the First Affiliated Hospital, Fujian Medical University, Fuzhou, 350212, China; c Fujian Provincial Institutes of Brain Disorders and Brain Sciences, The First Affiliated Hospital, Fujian Medical University, Fuzhou, 350005, China

**Keywords:** Dihydroartemisinin, Nanozyme, Chemodynamic therapy, Self-supplying, Ferroptosis

## Abstract

Chemodynamic therapy (CDT), leveraging intracellular iron ions (Fe^2+^) and hydrogen peroxide (H_2_O_2_), is a highly selective therapeutic strategy with significant potential. However, its clinical application is currently hindered by the limited catalytic activity of transition metal ions and insufficient H_2_O_2_ supply. In this study, we present a novel and effective CDT approach using an Fe single-atom nanozyme (Fe-SAE) to deliver dihydroartemisinin (DHA), a first-line antimalarial drug. DHA serves dual roles: as a substitute for H_2_O_2_ and as a recruiter of Fe^3+^, significantly enhancing the reactive oxygen species (ROS) cascade for self-amplified chemodynamic and ferroptosis therapy. Upon internalization by tumor cells, Fe-SAE, with its atomically dispersed active sites, exhibits remarkable peroxidase-like activity, catalyzing the generation of hydroxyl radicals from H_2_O_2_. Simultaneously, the endoperoxide bridge in released DHA is cleaved by Fe-SAE, further generating ROS and inducing lethal lipid peroxidation. DHA also upregulates the expression of transferrin receptor 1 (TfR1), facilitating Fe^3+^ influx and increasing intracellular Fe^3+^ levels, thereby enhancing chemodynamic efficacy. Additionally, Fe-SAE@D demonstrates glutathione oxidase-like activity, oxidizing reductive GSH to glutathione disulfide and promoting GPX4 inactivation. Both in vitro and in vivo studies confirm that Fe-SAE@D induces CDT and ferroptosis by self-supplying H_2_O_2_, initiating a ROS storm, and depleting glutathione. These synergistic effects significantly enhance CDT efficacy, presenting a promising strategy to overcome traditional CDT limitations.

## Graphical abstract

We present an effective CDT approach based on Fe single-atom nanozyme (Fe-SAE) that delivers the first-line antimalarial drug, dihydroartemisinin (DHA). DHA serves as both a substitute for hydrogen peroxide (H_2_O_2_) and a recruiter of Fe^3+^, significantly amplifying the reactive oxygen species (ROS) cascade reaction for self-amplified chemodynamic and ferroptosis therapy. Upon internalization by tumor cells, Fe-SAE, which features atomically dispersed active metal sites, demonstrates exceptional peroxidase-like activity, catalyzing the generation of hydroxyl radicals from H_2_O_2_. Concurrently, the endoperoxide bridge in the released DHA is cleaved by the highly active Fe-SAE, further generating ROS and inducing lethal lipid peroxidation. Furthermore, DHA acts as a recruiter by upregulating the expression of transferrin receptor 1, facilitating Fe^3+^ influx, increasing Fe^3+^ levels, and further enhancing chemodynamic efficacy. Furthermore, Fe-SAE@D also exhibits GSHOX-like activity, efficiently oxidizing reductive glutathione to glutathione disulfide, thereby inactivating glutathione peroxidase 4 and promoting ferroptosis.

**Figure:**
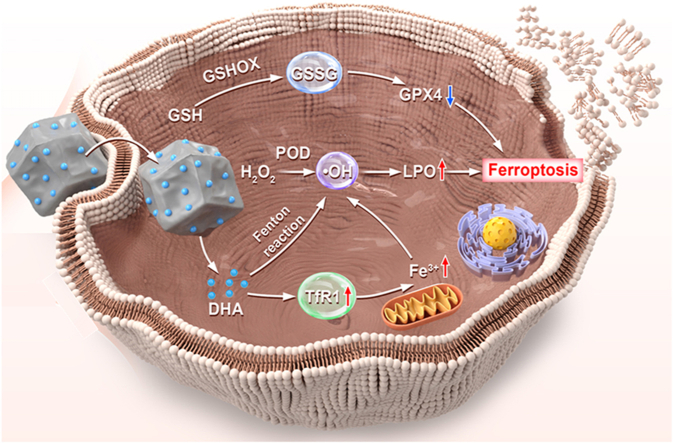

## 1 Introduction

Chemodynamic therapy (CDT) is a novel therapeutic approach that inhibits tumor growth by catalytically converting endogenous hydrogen peroxide (H_2_O_2_) into highly toxic hydroxyl radicals (·OH) [[1], [2], [3], [4]]. Unlike sonodynamic therapy, which relies on molecular oxygen (O_2_) and external ultrasound sources and is strongly inhibited by the hypoxic tumor microenvironment (TME), CDT avoids these major limitations through Fenton-type reactions that generate ·OH independently of O_2_ and ultrasound [[5], [6], [7], [8]]. To date, CDT has primarily employed low-valent transition metal ions to trigger ROS generation, thereby disrupting intracellular redox homeostasis and inducing tumor cell death [[9], [10], [11]]. However, despite the higher intracellular H_2_O_2_ levels in many tumor cells compared to noncancerous cells, the endogenously produced H_2_O_2_ is still insufficient for satisfactory chemodynamic efficacy [[12], [13], [14], [15]]. Additionally, high levels of reductive glutathione (GSH) in the TME and the relatively low Fenton activity of transition metal ions significantly restrict therapeutic efficacy [[16], [17], [18]]. Therefore, incorporating peroxide self-supply, ultra-high Fenton activity, and GSH depletion capacity into CDT nanoagents is highly desirable to enhance anticancer efficiency.

Dihydroartemisinin (DHA), a first-line antimalarial drug, has recently attracted significant attention as a promising antitumor agent against a wide range of tumor cells [19,20]. As a peroxide compound (ROOR) with an intramolecular peroxide bridge, DHA is particularly suitable for Fenton chemistry-mediated reactive oxygen species (ROS) generation [21]. Previous studies have demonstrated that the cytotoxicity of DHA can be significantly enhanced through the cleavage of its endoperoxide bridge by iron ions (Fe^2+^), leading to the efficient generation of highly toxic ·OH [22]. In this context, several strategies have been proposed to deliver DHA via Fe-based nanozymes for tumor therapy. For example, Li et al. developed an effective CDT strategy using DHA to substitute for H_2_O_2_ and recruit Fe^2+^, thereby significantly amplifying ROS generation for synergistic CDT-ferroptosis therapy [23,24]. However, the direct application of DHA is limited by its rapid degradation under physiological conditions and poor targeting efficiency [25,26]. Therefore, the delivery of DHA can efficiently overcome the deficiency of endogenous H_2_O_2_ and boost bioavailability, ultimately amplifying chemodynamic therapeutic efficacy.

Nanozymes, artificial nanomaterials that mimic enzyme activities, have become a focal point as alternatives to natural enzymes [[27], [28], [29], [30]]. Through precise molecular-level modulation of bio-catalytic sites, nanozymes offer key advantages such as lower costs, greater stability, and enhanced membrane permeability compared to natural enzymes [[31], [32], [33], [34]]. These benefits position nanozymes as highly promising tools for tumor diagnosis and therapy [35]. A wealth of evidence has demonstrated that nanozymes with peroxidase (POD)-like and glutathione oxidase (GSHOX)-like activities can induce oxidative stress by elevating ROS levels, thereby promoting tumor cell death [36,37]. However, several challenges remain despite their promising potential in tumor therapy [38]. The complex crystal structures and intricate surface configurations of nanozymes complicate the precise control of substrate selectivity and catalytic activity at the molecular level [39]. Moreover, the catalytic mechanisms of nanozymes remain poorly understood due to their complex nanostructures, diverse elemental compositions, and dynamic catalytic microenvironments [40]. A significant advancement in this field is the development of single-atom nanozymes (SAEs), which represent the next generation of nanozymes [[41], [42], [43], [44]]. SAEs are characterized by maximal atomic utilization, well-defined electronic and geometric structures, and enhanced catalytic activity, effectively bridging the gap between artificial and natural enzymes [[45], [46], [47], [48]]. These features not only boost intrinsic catalytic activity but also improve substrate selectivity. However, the high levels of reductive GSH in the TME inhibit sustained lipid peroxidation (LPO) accumulation, diminishing the efficacy of SAE-catalyzed ferroptosis. Therefore, strategies to deplete GSH in the TME to promote the ROS cascade reaction of SAEs are crucial for inducing irreversible ferroptosis and enhancing therapeutic outcomes.

**Scheme 1:**
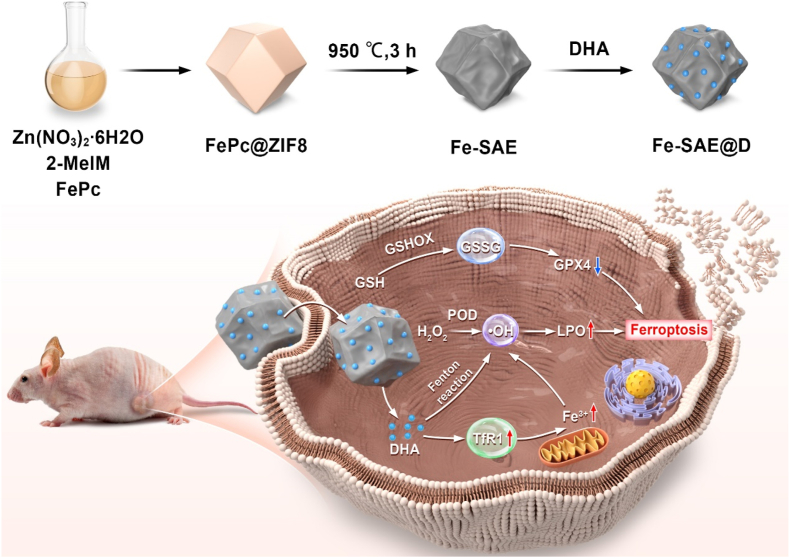
Schematic illustration of the anticancer mechanism of Fe-SAE@D.

Building on the foundational concept, we have engineered a prototype Fe-based SAE with Fe-N_4_ active sites, loaded with the antimalarial drug DHA, termed Fe-SAE@D, for enhanced chemodynamic and ferroptosis therapy (Scheme 1). The Fe-SAE structure consists of a nitrogen-doped carbon matrix that anchors isolated Fe^δ+^ ions (0 < δ < 2), each coordinated with four nitrogen atoms. The expanded graphite layer spacing maximizes the exposure of active sites, thereby boosting catalytic efficiency. Once internalized by cancer cells, Fe-SAE@D demonstrates POD-like activity, efficiently converting H_2_O_2_ into the highly toxic ·OH within the TME. Concurrently, the endoperoxide bridge in released DHA is cleaved by the active Fe-SAE, generating additional ROS and inducing lethal LPO. Moreover, DHA upregulates the expression of transferrin receptor 1 (TfR1), facilitating iron ion influx, raising Fe^3+^ levels, and further augmenting ROS generation. Crucially, Fe-SAE@D also exhibits GSHOX-like activity, oxidizing reduced GSH to GSSG, thereby inactivating GPX4 and promoting ferroptosis. Both in vitro and in vivo studies confirm that Fe-SAE@D effectively induces irreversible tumor ferroptosis through LPO accumulation, increased Fenton reaction substrates, and GPX4 inactivation. This research highlights the potential of a DHA-driven ROS cascade strategy for SAE-mediated TME regulation, significantly enhancing ferroptosis chemodynamic efficacy.

## 2 Results and discussion

**Fig. 1:**
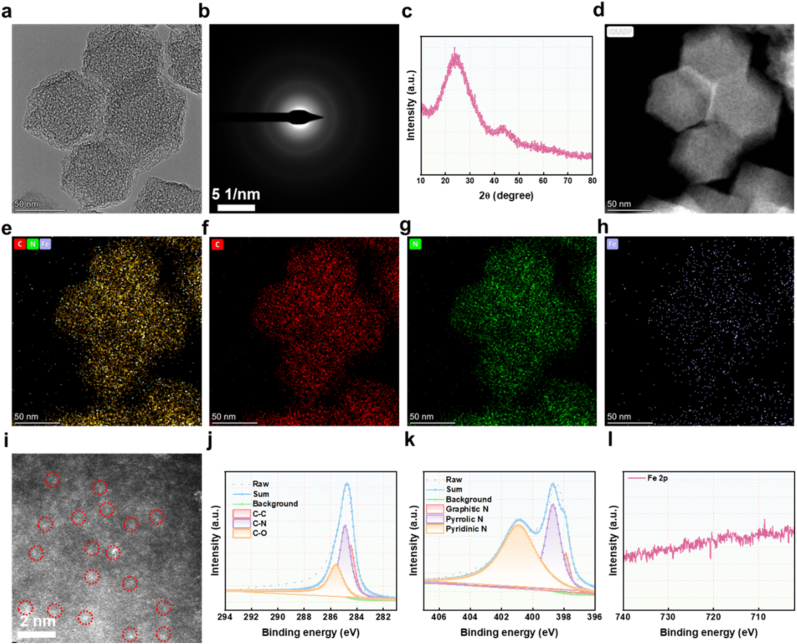
**Characterization of Fe-SAE.** (a) TME image of Fe-SAE. (b) SAED image of Fe-SAE. (c) XRD pattern of Fe-SAE. (d–h) EDX mapping image of Fe-SAE. (i) Ac HAADF-STEM image of Fe-SAE. Red circle indicates single-atom Fe. (j) High-resolution C 1s, (k) N 1s, and (l) Fe 2p spectra in Fe-SAE. (For interpretation of the references to color in this figure legend, the reader is referred to the Web version of this article.)

The synthesis of Fe-SAE was achieved through controlled carbonization of Fe@ZIF-8 precursors, as illustrated in Scheme 1. Initially, iron acetylacetonate (FePc) was encapsulated within ZIF-8 frameworks via a host-guest strategy, maintaining the characteristic rhombic dodecahedron morphology (Fig. S1). Subsequent pyrolysis of FePc@ZIF-8 yielded Fe-SAE with preserved polyhedral structures (Fig. 1a), exhibiting uniform sizes of ∼50 nm as revealed by transmission electron microscopy (TEM). Structural characterization showed significant morphological evolution during carbonization, with high-resolution TEM revealing the formation of a porous surface architecture (Fig. S2). The amorphous nature of the N-doped carbon matrix was confirmed by selected area electron diffraction (SAED) patterns showing diffuse diffraction rings (Fig. 1b), while X-ray diffraction (XRD) analysis excluded the presence of crystalline Fe or Fe oxide phases (Fig. 1c), strongly suggesting atomic dispersion of Fe species. Energy-dispersive X-ray spectroscopy (EDX) mapping (Fig. 1d–h, S3) demonstrated homogeneous distribution of Fe, C, and N throughout the material. Direct visualization of isolated Fe atoms was achieved through aberration-corrected atomic-resolution high-angle annular dark-field scanning transmission electron microscopy (Ac HAADF-STEM), where individual bright spots (red circles in Fig. 1i) corresponded to atomic Fe centers. Inductively coupled plasma mass spectrometry (ICP-MS) quantification determined the Fe loading at 0.85 wt%. Finally, hydrophobic loading of DHA yielded the functional composite Fe-SAE@D, completing the synthetic protocol. As shown in Figs. S5 and S6, we monitored the hydrodynamic diameter of Fe-SAE@D NPs when incubated in both blood and FBS over a 96-h period. The results demonstrate that the particle size remained remarkably stable and consistent (approximately between 130 nm and 150 nm) throughout the entire incubation. Furthermore, to directly assess potential iron leakage, we measured the Fe content in the blood samples co-incubated with Fe-SAE@D NPs over the same 96-h period, as presented in Fig. S6. The data clearly shows that the Fe content in the blood remained consistently stable at approximately 0.8 %–0.9 % throughout the prolonged incubation. This indicates excellent colloidal stability of our Fe-SAE nanoparticles in complex physiological environments, preventing aggregation or significant degradation. These results confirm the successful fabrication of Fe-SAE@D.

**Fig. 2:**
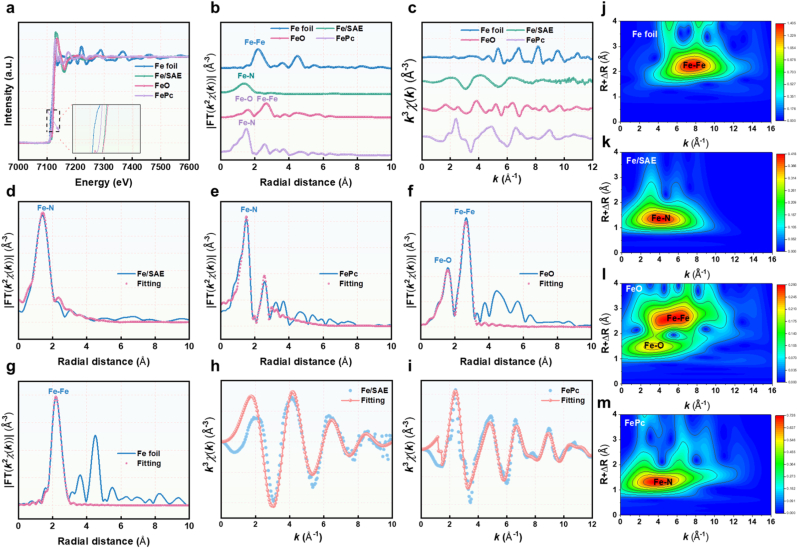
**Atomic structural analysis of Fe-SAE.** (a) Fe K-edge XANES spectra of Fe foil, FePc, FeO, and Fe-SAE (inset: the enlarged pre-edge region). (b) The Fourier transform EXAFS of the Fe K-edge of Fe foil, FePc, FeO, and Fe-SAE. (c) EXAFS curves of Fe foil, FePc, FeO, and Fe/SAE at the k space. (d) EXAFS fitting curves of Fe-SAE, (e) Fe foil, (f) FePc, and (g) FeO at the R space. (h) EXAFS fitting curve of Fe-SAE and (i) Fe foil at the k space. (j) Wavelet transformation of Fe K-edge EXAFS of Fe foil, (k) Fe-SAE, (l) FeO, and (m) FePc.

X-ray photoelectron spectroscopy (XPS) was employed to probe the chemical states of Fe, C, and N in Fe-SAE. The high-resolution C 1s spectrum (Fig. 1j) displayed dominant sp^2^-hybridized graphitic carbon contributions, including C-C, C-N, and C-O bonds. Deconvolution of the N 1s spectrum identified three nitrogen configurations-graphitic N, pyridinic N, and pyrrolic N-with the latter providing key coordination sites for stabilizing single-atom Fe centers, thereby enhancing catalytic activity. The Fe 2p_3/2_ peak at 715.5 eV, situated between Fe^0^ (710.5 eV) and Fe^2+^ (725.3 eV), confirmed an intermediate oxidation state (Fe^δ+^, 0 < δ < 2) (Fig. 1k). To further elucidate the Fe coordination environment, synchrotron-radiation X-ray absorption spectroscopy (XAS) was performed. The Fe K-edge XANES spectrum exhibited an absorption edge energy between Fe foil and FeO (Fig. 2a), corroborating the Feδ^+^ state observed in XPS. Fourier-transform EXAFS analysis revealed a prominent peak at 1.38 Å, assigned to Fe–N scattering, while the absence of a peak at 2.67 Å (characteristic of Fe-Fe bonds) confirmed the atomic dispersion of Fe (Fig. 2b and c). Quantitative EXAFS fitting indicated a Fe-N_4_ coordination structure (Fig. 2d–i, S7, and Table S1). Wavelet transform (WT) analysis further validated the absence of Fe–Fe contributions, with a singular intensity maximum at 4.5 Å^−1^ corresponding to Fe–N coordination (Fig. 2j–m). These results unambiguously establish the successful synthesis of Fe-SAE with atomically dispersed Fe-N_4_ active sites.

**Fig. 3:**
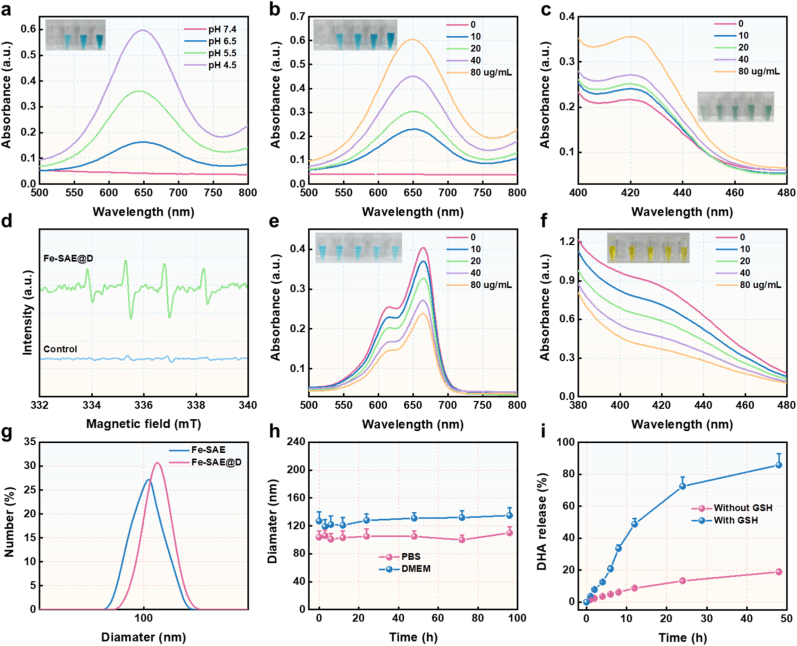
**Enzymatic performance of Fe-SAE.** (a) UV–vis spectra of TMB incubated with Fe-SAE and H_2_O_2_ at different pH. (b) UV–vis spectra of TMB treated with different concentrations of Fe-SAE in the presence of H_2_O_2_. (c) The UV–vis spectra of ABTS treated with different concentrations of Fe-SAE in the presence of H_2_O_2_. (d) The ESR curves of •OH captured using a DMPO. (e) The UV–vis spectra of MB treated with different concentrations of Fe-SAE in the presence of H_2_O_2_. (f) UV–vis spectra of DTNB treated with different concentrations of Fe-SAE in the presence of GSH. (g) The DLS results of Fe-SAE and Fe-SAE@D. (h) The biostability of Fe-SAE@D in PBS and DMEM. (i) The DHA release curves in the presence of GSH.

After determining the coordination number of Fe atoms in Fe-SAE, we systematically evaluated its enzymatic activities, including its POD- and GSHOX-mimicking activities. The 3,3′,5,5′-tetramethylbenzidine (TMB) assay was used to quantify the POD-mimicking activity of Fe-SAE. Upon treatment with H_2_O_2_, a rapid color change in TMB was observed at acidic pH (ranging from 4.3 to 6.5), but not at neutral pH (7.4). This indicates the pH-dependent catalytic behavior of Fe-SAE and its efficient conversion of H_2_O_2_ into •OH under mildly acidic conditions (Fig. 3a and S8). Moreover, the catalytic performance of Fe-SAE demonstrated a clear concentration-dependent behavior (Fig. 3b and S9). To verify •OH generation, we employed 2,2′-azino-bis(3-ethylbenzothiazoline-6-sulfonic acid) (ABTS), which exhibited a pronounced absorbance increase upon H_2_O_2_ treatment, confirming Fe-SAE's robust POD-like activity (Fig. 3c and S10). Further validation using electron spin resonance (ESR) spectroscopy with dimethyl-1-pyrroline N-oxide (DMPO) as a spin trap revealed the characteristic 1:2:2:1 quartet signal of the DMPO/•OH adduct under acidic conditions (Fig. 3d). The •OH production efficiency was further assessed via methylene blue (MB) degradation assays, where a concentration-dependent reduction in absorbance was observed upon incubation with Fe-SAE and H_2_O_2_ (Fig. 3e and S11). We conducted a TMB assay comparing Fe-SAE with other reported nanozymes (CuO, V_2_O_5_, Fe_3_O_4_). As shown in Fig. S12, Fe-SAE exhibited significantly higher POD-like activity, indicating its superior catalytic efficiency. We determined the Michaelis constant (Km) for Fe-SAE and compared it with other reported nanozymes. As shown in Fig. S13, Fe-SAE exhibited a significantly lower Km value, indicating its superior substrate affinity and catalytic efficiency. This data is now included in the revised manuscript. Additionally, Fe-SAE exhibited GSHOx-like activity, as evidenced by the decreasing absorbance at 412 nm in 5,5′-dithiobis-(2-nitrobenzoic acid) (DTNB) assays, indicating effective GSH depletion (Fig. 3f and S14). Dynamic light scattering (DLS) measurements confirmed the hydrodynamic size of Fe-SAE and Fe-SAE@D to be ∼100 nm (Fig. 3g). Notably, Fe-SAE@D displayed excellent stability in DMEM and PBS (Fig. 3h), while GSH-triggered DHA release was facilitated by the redox-responsive DSPE-S-S-PEG linkage (Fig. 3i and S15). Collectively, these findings underscore Fe-SAE's exceptional catalytic properties and its potential to amplify ROS cascades.

**Fig. 4:**
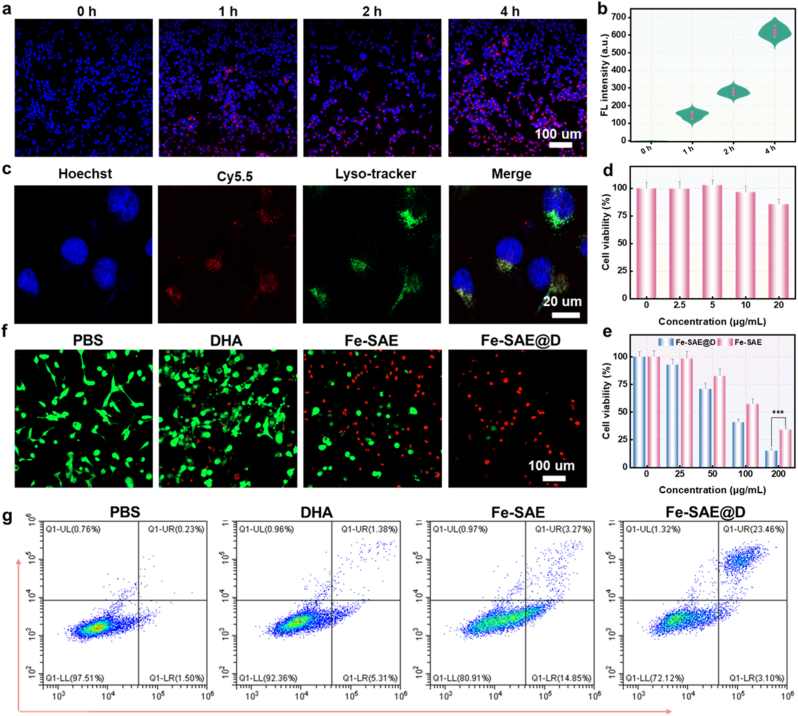
**The therapeutic efficacy on tumor cells.** (a) The CLSM images and (b) corresponding quantification of cancer cells treated with Cy5.5-labeled Fe-SAE@D.(c) The CLSM images of cancer cells colocalization. (d) Noncancerous and (e) tumor cell viability after 24 h of incubation with varying concentrations of Fe-SAE@D and Fe-SAE. (f) The fluorescence images of live/dead staining for cancer cells incubated with various formulations. (g) Flow cytometry measurements of cancer cells after incubation with different formulations. ∗∗∗P < 0.001.

To evaluate the cytotoxicity of Fe-SAE@D, its impact on GL261 cells was investigated. For cellular uptake experiments, Fe-SAE@D was labeled with cyanine 5.5 (Cy5.5). Confocal laser scanning microscopy (CLSM) observations revealed a time-dependent increase in red fluorescence within GL261 cells, indicating the accumulation of Fe-SAE@D (Fig. 4a and b, and S16). Colocalization studies suggested that Fe-SAE@D predominantly localized in lysosomes and the cytoplasm (Fig. 4c), aligning with the acidic conditions necessary to enhance the Fe-SAE@D-mediated Fenton reaction. As shown in Fig. S17, Fe-SAE@D NPs significantly increased intracellular iron accumulation, which is crucial for its ferroptosis-inducing mechanism. The cytotoxic effects of Fe-SAE@D on GL261 cells were assessed using the Cell Counting Kit-8 (CCK-8) assay (Fig. S18). As depicted in Fig. 4e, the survival rate of GL261 cells decreased markedly with escalating Fe-SAE@D concentrations. Fe-SAE@D demonstrated a more pronounced inhibitory effect than Fe-SAE, achieving an 85.04 % reduction in cell survival at 200 μg/mL, compared to 66.13 % inhibition with Fe-SAE. To further scrutinize the antitumor efficacy of Fe-SAE@D, a propidium iodide (PI)/calcein-AM co-staining assay was conducted. The results indicated that Fe-SAE@D generated stronger red fluorescence intensity than Fe-SAE, signifying a higher extent of cell death (Fig. 4f and S19). Flow cytometry analysis using Annexin V-FITC and PI staining further corroborated the anti-proliferative impact of Fe-SAE@D (Fig. 4g and S20), with the highest proportion of cell death observed in the Fe-SAE@D group. Collectively, these findings underscore that Fe-SAE@D outperforms Fe-SAE in anti-proliferative effects, capitalizing on its self-supplied H_2_O_2_ and cascade catalytic activities.

**Fig. 5:**
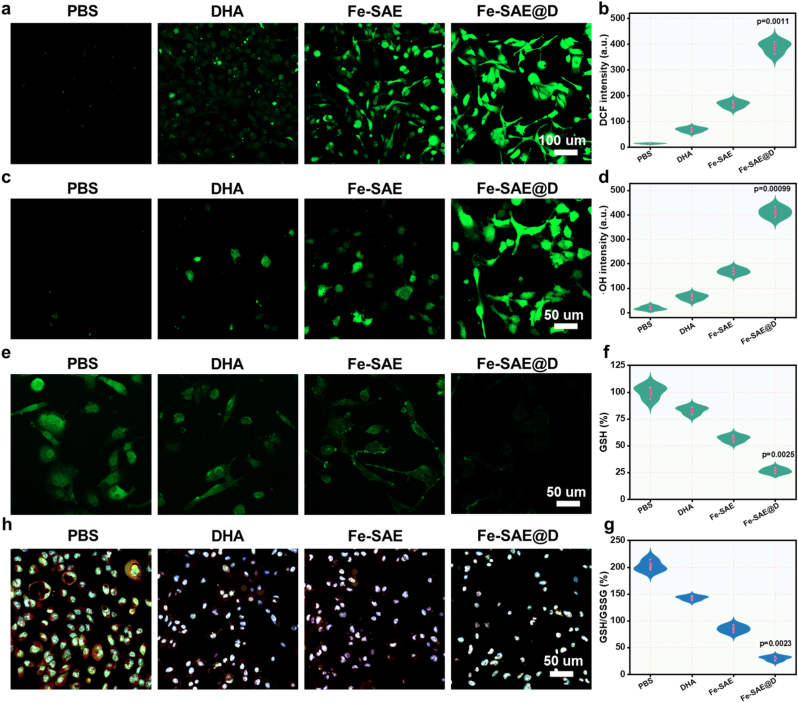
**The ROS generation in tumor cells.** (a) DCF fluorescence images and (b) corresponding quantification of cancer cells following varying treatments. (c) Fluorescence images and (d) corresponding quantification of cancer cells following varying treatments. (e) GSH fluorescence images of cancer cells following varying treatments.(f) The DTNB measurements of GSH levels and (g) GSH/GSSG rate exposed various formulations. (h) CLSM images of AO-stained GL261 cells after 24 h of incubation with different formulations.

To fully elucidate the therapeutic impact of Fe-SAE@D, its anti-tumor mechanisms were meticulously explored. Leveraging the peroxidase-like activity of Fe-SAE@D and its capacity for self-supplying H_2_O_2_, the fluorescent probe 2′,7′-dichlorofluorescein diacetate (DCFH-DA) was used to measure ROS levels in GL261 cells. The findings revealed that Fe-SAE@D markedly increased ROS levels, with DHA further boosting ROS accumulation in these cells (Fig. 5a and b, and S21). To specifically evaluate the generation of intracellular ·OH, the ·OH-specific fluorescent probe O27 was employed. CLSM images confirmed that Fe-SAE@D was highly effective in promoting ·OH production (Fig. 5c and d, and S22), which significantly amplified the overall ROS levels. Additionally, intracellular GSH is crucial for buffering ROS levels. Notably, Fe-SAE@D demonstrated a remarkable ability to deplete GSH, inducing oxidative stress and triggering a ROS storm within the cells (Fig. 5e–g and S23). Moreover, Fe-SAE@D-mediated ·OH formation in cancer cells led to the damage of cellular organelles, including lysosomes (Fig. 5h and S24). Collectively, these results confirm that the ROS generation, self-supplied H_2_O_2_, and GSH depletion capacities of Fe-SAE@D play a substantial role in inducing tumor cell death.

**Fig. 6:**
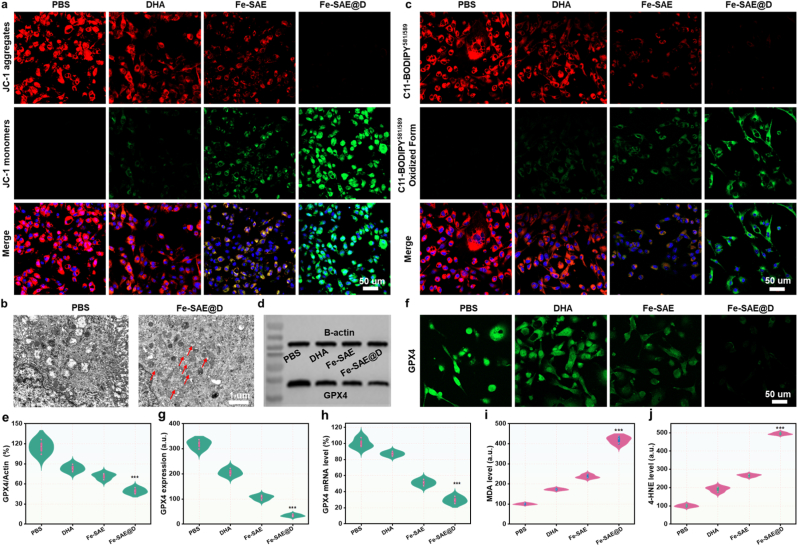
**Mechanism of Fe-SAE@D induced cancer cells ferroptosis.** (a) JC-1 fluorescence images of tumor cells following different treatments. (b) Bio-TEM of tumor cells following various treatments. (c) The confocal images of fluorescent probe C11-BODIPY^581/589^-stained cancer cells following various treatments. (d) GPX4 expression and (e) corresponding quantification in cancer cells incubated with varying formulations by WB. (f) The GPX4 expressions and (g) corresponding quantification of cancer cells following varying treatments by immunofluorescence assay. (h) The GPX4 mRNA level of cancer cells following varying treatments. (i) MDA and (j) 4-HNE levels in tumor cells following various treatments. ∗∗∗P < 0.001.

To assess mitochondrial membrane potential polarization, we utilized the mitochondria-specific probe JC-1, which detects shifts in fluorescence from red to green. CLSM images (Fig. 6a and S25) revealed significantly higher green fluorescence intensity in the Fe-SAE group compared to the PBS group. The presence of DHA further intensified the green fluorescence in the Fe-SAE@D group, while red fluorescence intensity showed an inverse trend. Additionally, biological transmission electron microscopy (Bio-TEM) analysis of tumor cells treated with Fe-SAE@D unveiled mitochondrial damage, a hallmark of ferroptosis (Fig. 6b). These results suggest that Fe-SAE@D treatment induces mitochondrial membrane depolarization in GL261 cells, potentially triggering ferroptosis. We further examined key indicators of ferroptosis. Highly reactive ·OH can oxidize polyunsaturated fatty acids, leading to LPO accumulation. To monitor LPO in cell membranes, we employed the fluorescent indicator C11-BODIPY^581/589^. Fe-SAE treatment significantly increased green fluorescence while gradually reducing red fluorescence, indicating LPO accumulation, an effect further amplified by DHA (Fig. 6c and S26). Concurrently, we evaluated GPX4 expression levels in GL261 cells. Western blot (WB) analysis (Fig. 6d and e) showed that Fe-SAE@D treatment downregulated GPX4 expression, a finding supported by immunofluorescence and quantitative real-time polymerase chain reaction (qRT-PCR) data (Fig. 6f–h and S27). In addition, we performed immunofluorescence staining to observe TFR1 expression in GL261 cells under different drug interventions. As shown in Fig. S28, the DHA can significantly increase the TFR1 expression in tumor cells, thereby enhancing the uptake of Fe ions. Moreover, we assessed additional ferroptosis markers, including malondialdehyde (MDA) and 4-hydroxynonenal (4-HNE). Fe-SAE combined with DHA significantly elevated MDA and 4-HNE levels in GL261 cells (Fig. 6i and j). Collectively, these results demonstrate that Fe-SAE@D induces ferroptosis in tumor cells through LPO accumulation and GPX4 inactivation.

**Fig. 7:**
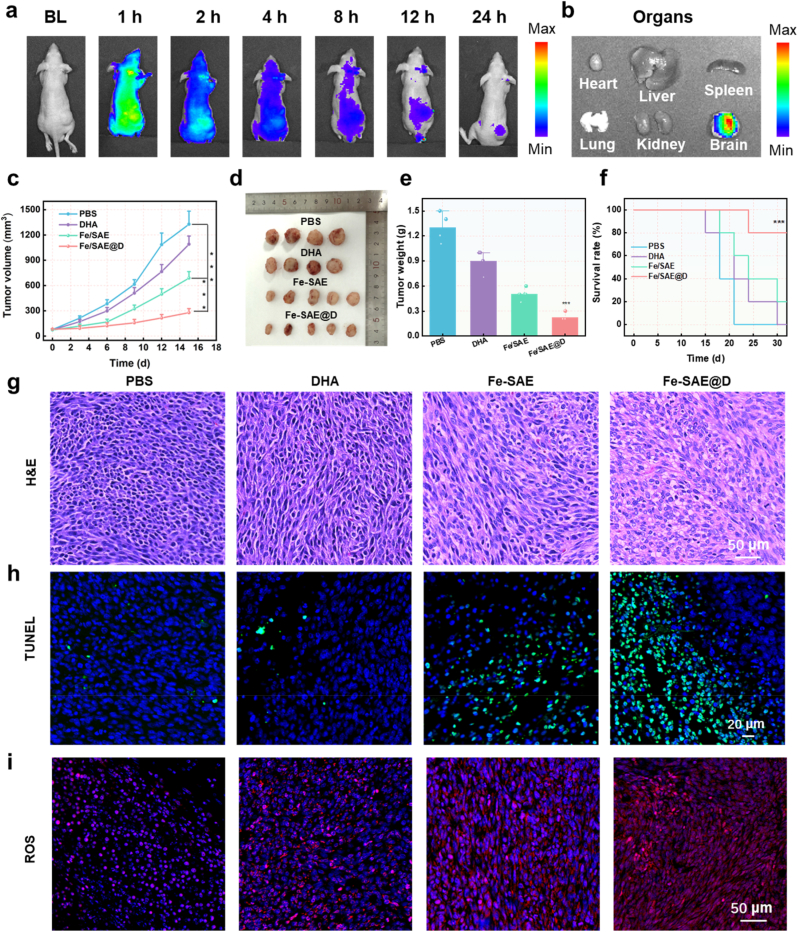
**Antitumor efficacy of Fe-SAE@D.** (a) Fluorescence images of tumor-bearing mice at various time points following injection with Cy5.5-labeled Fe-SAE@D. (b) Fluorescence images of the major organs (heart, liver, spleen, lung, kidney, brain) and tumor taken from the tumor bearing mice at 24 h after intravenous injection. (c) The tumor volume curves of mice following various formulations. (d) The photographs and (e) tumor weights of mice following different formulations. (f) The Kaplan-Meier survival curves of mice following various formulations. (g) H&E staining, (h) TUNEL immunofluorescence, and (i) ROS immunofluorescence of tumors following various formulations. ∗∗∗P < 0.001.

Animal experiments were conducted in strict accordance with the protocol approved by the Ethical Committee of Fujian Medical University (IACUC FJMU2022-0608). Before assessing the therapeutic efficacy of Fe-SAE@D in mice, its biosafety was rigorously evaluated. Hematoxylin and eosin (H&E) staining was used to examine the normal cellular morphology of various tissues, with results showing no significant histological damage (Fig. S29). We have evaluated the biosafety of Fe-SAE@D NPs by analyzing key blood biochemical indicators, hemolysis test and routine blood test in mice. Results showed no significant changes in the blood biochemical indicators of mice injected with Fe-SAE@D NPs over 15 days, indicating good biocompatibility and no obvious toxicity (Figs. S30–32). To explore the in vivo tumor accumulation of Fe-SAE@D, an IVIS imaging system was employed. Fluorescence imaging revealed that the fluorescence signal of Cy5.5-labeled Fe-SAE@D in the tumor region progressively intensified and remained at a high level for up to 24 h post-injection. This indicates substantial tumor accumulation of Fe-SAE@D, likely due to the enhanced permeability and retention (EPR) effect (Fig. 7a and b and S33). The in vivo antitumor efficacy of Fe-SAE@D was further investigated, leveraging its DHA delivery capabilities and remarkable catalytic activities. GL261-bearing nude mice were randomly divided into four treatment groups: Control, DHA, Fe-SAE, and Fe-SAE@D. Body weight measurements showed no significant changes in the Fe-SAE and Fe-SAE@D groups compared to the PBS group (Fig. S34), further confirming the excellent biocompatibility of Fe-SAE@D. Tumor growth progression was closely monitored, with the results shown in Fig. 7c. Fe-SAE treatment, which induces ferroptosis via GPX4 inactivation and LPO accumulation, partially inhibited tumor growth. Notably, the Fe-SAE@D group exhibited a more pronounced antitumor effect, attributed to its unique DHA-enhanced ROS cascade (Fig. 7d and e). Kaplan-Meier survival analysis revealed that Fe-SAE@D significantly improved survival rates, demonstrating substantial therapeutic efficacy against tumors (Fig. 7f). The antitumor effect of Fe-SAE@D was further validated through H&E and TUNEL staining of tumor sections. H&E staining revealed extensive tumor tissue damage in mice treated with Fe-SAE@D, highlighting its potent therapeutic effect (Fig. 7g). TUNEL staining indicated the highest levels of cell death in the Fe-SAE@D treatment group (Fig. 7h and S35). Immunofluorescence staining further demonstrated a significant reduction in GPX4 expression in tumor cells following Fe-SAE@D treatment (Fig. S36). ROS levels were markedly elevated in the tumor cells of the Fe-SAE@D-treated group, attributed to the DHA-mediated self-supplying H_2_O_2_ and the POD- and GSHOX-like catalytic activities of Fe-SAE (Fig. 7i). Collectively, these findings highlight the role of Fe-SAE@D in self-supplying H_2_O_2_, generating •OH, depleting GSH, and promoting LPO accumulation, ultimately leading to tumor ferroptosis.

## 3 Conclusion

In conclusion, we have successfully engineered the Fe-SAE system as a novel DHA delivery platform to enhance ROS-driven ferroptosis in cancer cells. Fe-SAE, with its atomically dispersed active metal sites, demonstrates robust catalytic activities similar to POD and GSHOX, effectively promoting •OH generation from H_2_O_2_ and intracellular GSH depletion. The endoperoxide bridge in released DHA can be efficiently cleaved by the highly active Fe-SAE, further generating ROS and triggering lethal LPO. Additionally, DHA upregulates TfR1 expression, facilitating iron ion influx, increasing intracellular Fe^3+^ levels, and amplifying ROS production. Fe-SAE@D also effectively depletes endogenous reductive GSH, further activating GPX4 inactivation. Comprehensive in vitro and in vivo studies have elucidated the mechanism of tumor cell death mediated by Fe-SAE@D, highlighting its dual role in DHA delivery, ROS cascade initiation, and reductive GSH depletion, all of which synergistically induce CDT-driven ferroptosis. This research demonstrates significant potential for advancing the clinical application of DHA as an antitumor agent, offering a novel and effective strategy for cancer treatment.

## CRediT authorship contribution statement

**Yuanlong Zhang:** Conceptualization. **Binghong Chen:** Data curation. **Penghui Wei:** Formal analysis. **Zhongyuan Shen:** Investigation. **Xiyue Wu:** Resources. **Wenzhong Mei:** Supervision. **Yang Zhu:** Writing – review & editing, Writing – original draft, Visualization, Supervision. **Yuanxiang Lin:** Visualization, Supervision.

## Ethics approval and consent to participate

Animal experiments were performed according to the protocol approved by The Ethical Committee of Fujian Medical University (IACUC FJMU2022-0608).

## Consent for publication

All authors agreed to submit this manuscript.

## Funding

This work was supported by 10.13039/501100017686Fujian Provincial Health Technology Project (Grant number 2024CXA017), the Joint Funds for the innovation of science and Technology, Fujian province (Grant number: 2023Y9090, 2024Y9172), National Clinical Key Specialty (Neurosurgery) Project of China, 10.13039/501100003392Natural Science Foundation of Fujian Province (2023J05132).

## Declaration of competing interest

The authors declared that they have no known competing financial interests or personal relationships that could have appeared to influence the work reported in this paper.

## Data Availability

Data will be made available on request.
