# Supplementary material for: Single-atom nanozyme-mediated dihydroartemisinin delivery for self-enhanced chemodynamic therapy and ferroptosis
Source: Mater Today Bio. 2025 Jul 22;34:102096. doi: 10.1016/j.mtbio.2025.102096 (PMC12320232; doi:10.1016/j.mtbio.2025.102096)
Supplement: Multimedia component 1 [file mmc1.docx]

**Supporting Information**

**Chemicals**

2,2’-azino-bis(3-ethylbenzothiazoline-6-sulfonic acid) diammonium salt (ABTS), ironylacetonate (Fecac)_3_), hydrogen peroxide (H_2_O_2_), acetic acid (HAc), sodium acetate (NaAc), and ethanol were purchased from Sinopharm Chemical Reagents (Shanghai, China). 3,3',5,5'-tetramethylbenzidine (TMB), dihydroartemisinin (DHA) and C11-BODIPY^581/591^ were provided by Sigma-Aldrich (St. Louis, USA). Hoechst 33342, 2′,7′-dichlorofluorescin diacetate (DCFH-DA), cell count kit-8 (CCK-8), annexin V-FITC/PI apoptosis detection kit, thiobarbituric acid (TBA), and 1,1',3,3'-tetraethyl-5,5',6,6'-tetrachloroimidacarbocyanine iodide (JC-1) were bought from Beyotime (Shanghai, China). Dulbecco's modified eagle medium (DMEM) was purchased from Hyclone (Logan, USA). 5,5-dimethyl-1-pyrroline N-oxide (DMPO) was bought from Dojindo (Dojindo). Cyanine 5.5 monosuccinimidyl ester (Cy5.5-NHS), ELISA, and annexin V-FITC/PI apoptosis detection kit were purchased from Beijing Solarbio Science & Technology Co., Ltd. (Beijing, China). Live & Dead Staining Kit (Cat#40274ES60) and GMyc-PCR Mycoplasma Test Kit (Cat#40601) was purchased from Yeasen Biotechnology (Shanghai) Co., Ltd.. 20 mm glass-bottom dishes, and centrifuge tubes were obtained from NEST Biotechnology Co. Ltd. (Wuxi, China). Deionized (DI) water was obtained from a Milli-Q water purification system.

**Instruments**

Powder X-ray diffraction (XRD) patterns were recorded on a Rigaku Miniflex-600 diffractometer. Transmission electron microscope (TEM) images were taken by Hitachi-7700. High-angle annular dark field scanning transmission electron microscopy (HAADF-STEM) images were recorded by JEM-ARM200F (JEOL) TEM/STEM with a spherical aberration corrector. The energy-dispersive X-ray spectroscopy (EDS) mapping was performed by JEM-2100F. X-ray photoelectron spectroscopy (XPS) spectra were collected on scanning X-ray microprobe (PHI 5000 Verasa, ULAC-PHI). Scanning electron microscopy (SEM) images were taken by Nova NanoSEM 230. Fluorescence imaging was performed by confocal microscopy (Nikon C2). The absorption spectra were measured by a ultraviolet-visible (UV-vis) UH4150 spectrophotometer (Hitachi). Metal content was measured by using inductively coupled plasma mass spectrometer (ICP-MS, PlasmaQuad 3, Thermo Elemental). Hydrodynamic diameters and zeta potentials were determined by a Zetasizer nano ZS instrument (Malvern). Cancer cell apoptosis was monitored by using a flow cytometer (CytoFLEX, Beckman).

**Experimental section**

**Synthesis of Fe-SAE**

Fe@ZIF-8 precursors were synthesized using a host-guest strategy. 4 g 2-methylimidazole and 100 mg Fe(acac)_3_ were added into a 150 mL flask containing 60 mL methanol as solution A. 2 g Zn(NO_3_)_2_•6H_2_O was dissolved in 60 mL methanol as solution B. Then solution A was mixed with solution B, and the mixture was further stirred for 24 h. Then the Fe@ZIF-8 powders were collected by centrifugation, washed with methanol several times, and dried at 65 °C in a vacuum oven overnight. And the Fe-SAE was obtained by the one-step pyrolysis strategy. Firstly, the Fe@ZIF-8 powder were placed successively in a tubular furnace, and then pyrolysis at 950 ºC for 3 h in an argon atmosphere. 10 mg Fe-SAE and 10 mL 40 mg/mL DSPE-S-S-PEG were mixed and ultrasound for 20 min, then 1.5 mL 1 mg/ mL DHA (in DMSO) was added. the mixture was further stirred for 12 h and the product Fe-SAE@D were collected by centrifugation and washed three time with PBS.

**•OH generation by Fe-SAE-mediated catalytic reaction**

The catalytic activity of Fe-SAE were measured by using TMB as a probe which can be converted into oxidized TMB with blue color by •OH. Briefly, Different concentrations of Fe-SAE and H_2_O_2_ (100 μM) were successively added into the PBS solutions with TMB (50 ug/mL), and the mixtures were shaken at 37 °C for 10 min. After centrifugation, the absorption spectra of supernatant were measured.

Fe-SAE and H_2_O_2_ (10 mM) were mixed with MB (20 ug/mL) solutions at acidic pH, and the mixtures were shaken at 37 °C for 10 min. After ultrafiltration, the •OH-induced MB degradation was measured by the absorbance change at 652 nm.

Electron spin resonance (ESR) analysis was carried out using DMPO as the spin trapper. To confirm the Fe-SAE-mediated •OH generation. 10 mM NaAc-HAc buffer solution (pH 4.3) containing 5 mM H_2_O_2_, Fe-SAE (50 μg/mL), and 100 mM DMPO was ultrasonicated for 1 min. Then, the mixture was transferred to a quartz tube for ESR measurement.

**GSH consume by Fe-SAE mediated catalytic reaction**

Fe-SAE were mixed with GSH (10 mM) solutions at acidic pH, and the mixtures were shaken at 37 °C for 30 min. Finally, the DTNB (0.5 mg/ml) solution was added. After ultrafiltration, the GSH depletion was measured by the absorbance change at 415 nm.

**Cytotoxicity assessments**

Cell-viability was determined by the CCK-8 assay. For CCK-8 assay, GL261 cells were planted for 24 h. Then, the cells were incubated with various concentrations of Fe-SAE@D. After treatment for 24 h, the medium was replaced with fresh medium containing 10 μL CCK-8 and quantified by the absorbance at 450 nm using a microplate reader. For analysis of cell death, Annexin V-FITC and PI kit was conducted. GL261 cells were seeded and incubated 12 h. Subsequently, the cells were exposed to Fe-SAE@D. After co-staining with Annexin V-FITC and PI according to the manufacturer’s protocols. The quantitative cell death was analyzed by flow cytometry.

**In vitro ROS generation**

GL261 cells were seeded in confocal dish. After incubation for 12 h, the cells were treated with various formulation for 4 h. Then, the cells were co-stained with DCFH-DA (10 μM) and Hoechst (20 μM). After 20 minutes of incubation, the fluorescence imaging of cells was imaged by confocal microscopy.

**Analysis of the change of mitochondrial membrane potential (MMP)**

To investigate the MMP, GL261 cells were seeded and incubated for 24 h. Subsequently, the cells were exposed to Fe-SAE@D. Then the cells were treated according to the JC-1 kit. The fluorescence imaging of cells was analyzed by confocal microscopy.

**LPO initiated by Fe-SAE@D**

The cellular LPO assay was carried out by using a BODIPY^581/591^-C11 probe. GL261 cells were seeded and incubated for 24 h. Subsequently, the cells were exposed to Fe-SAE@D. Then the cells were stained with BODIPY^581/591^-C11 probe and Hoechst for 20 min. The fluorescence imaging of cells was imaged by CLSM.

**Analysis of the change of MDA**

To investigate the MDA, GL261 cells were seeded and incubated for 24 h. Subsequently, the cells were exposed to Fe-SAE@D. Then the cells were treated according to the MDA kit. The assay was carried out according to the manufacturer’s instructions.

**In vivo antitumor efficacy**

The right hind legs of all mice were subcutaneously transplanted with GL261 cells (1 × 10^6^ cells suspended in 100 μL of PBS). The tumor-bearing mice were used for antitumor treatment until the tumor volume reached about 70 mm^3^. GL261 tumor-bearing nude mice were randomly divided into 4 groups (5 mice per group): Fe-SAE@D, Fe-SAE, DHA or PBS. The mice were administered 5 mg/kg intravenously every two days for a total of three doses. The tumor volumes and body weights were recorded every three days. After 15 days of treatment, one mice from each group were euthanatized for histological examination and immunofluorescence.

**Statistical analysis**

All quantitative data were expressed as the mean ± standard deviation (SD). Statistical analyses were performed using the Student’s two-tailed t-test (*P < 0.05, **P < 0.01, ***P < 0.001,).

**
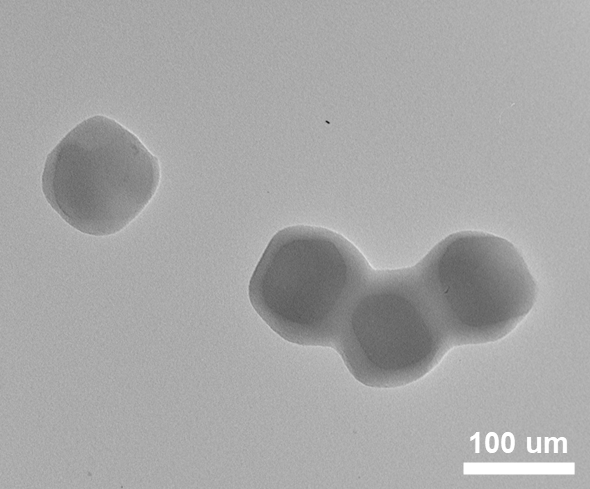
**

**Figure S1.** The TEM images of Fe@ZIF-8.


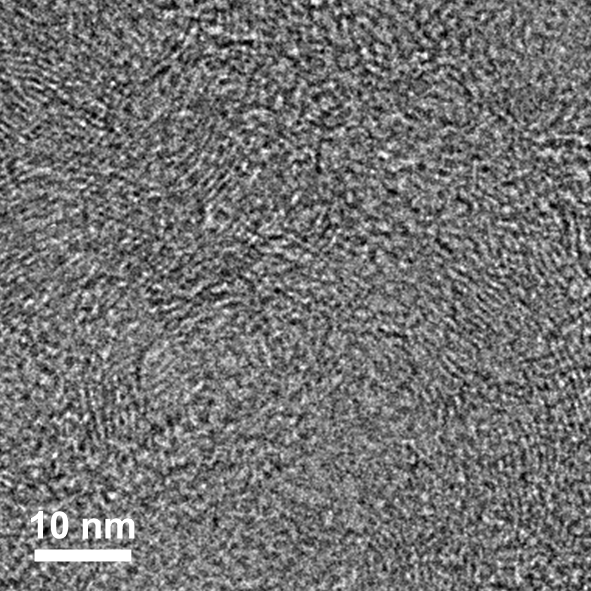


**Figure S2.** The HR-TEM images of Fe-SAE.


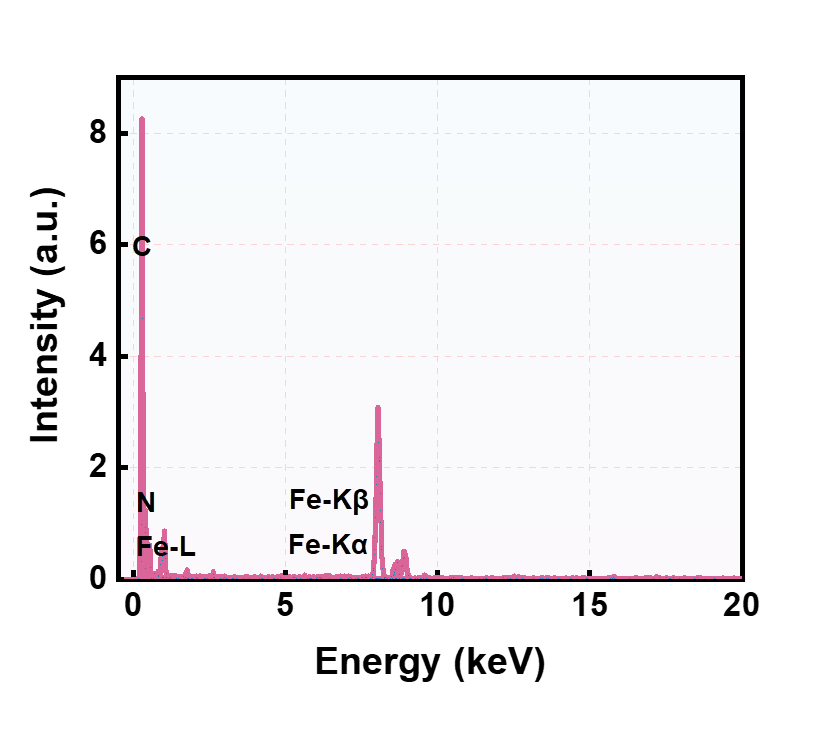


**Figure S3.** The EDX spectrum of Fe-SAE.


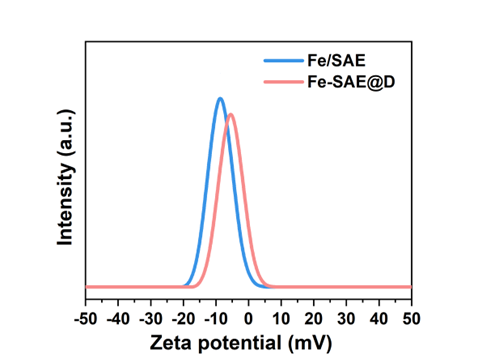


**Figure S4**. Zeta potential distribution of Fe/SAE and Fe-SAE@D NPs.


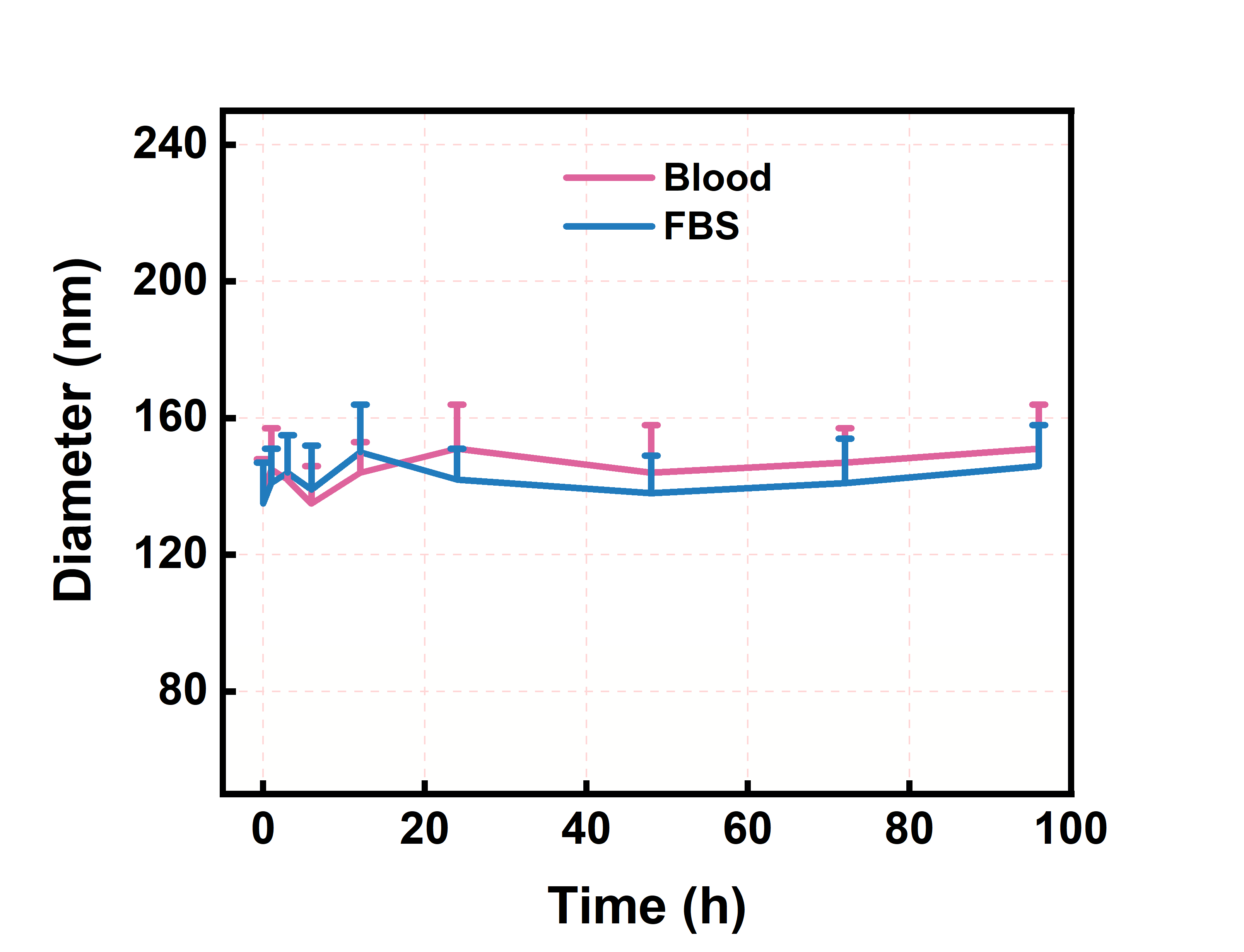


**Figure S5.** Hydrodynamic diameter of Fe-SAE@D NPs) incubated in blood and fetal bovine serum (FBS) over 96 hours. Data are presented as mean ± standard deviation (n=3).


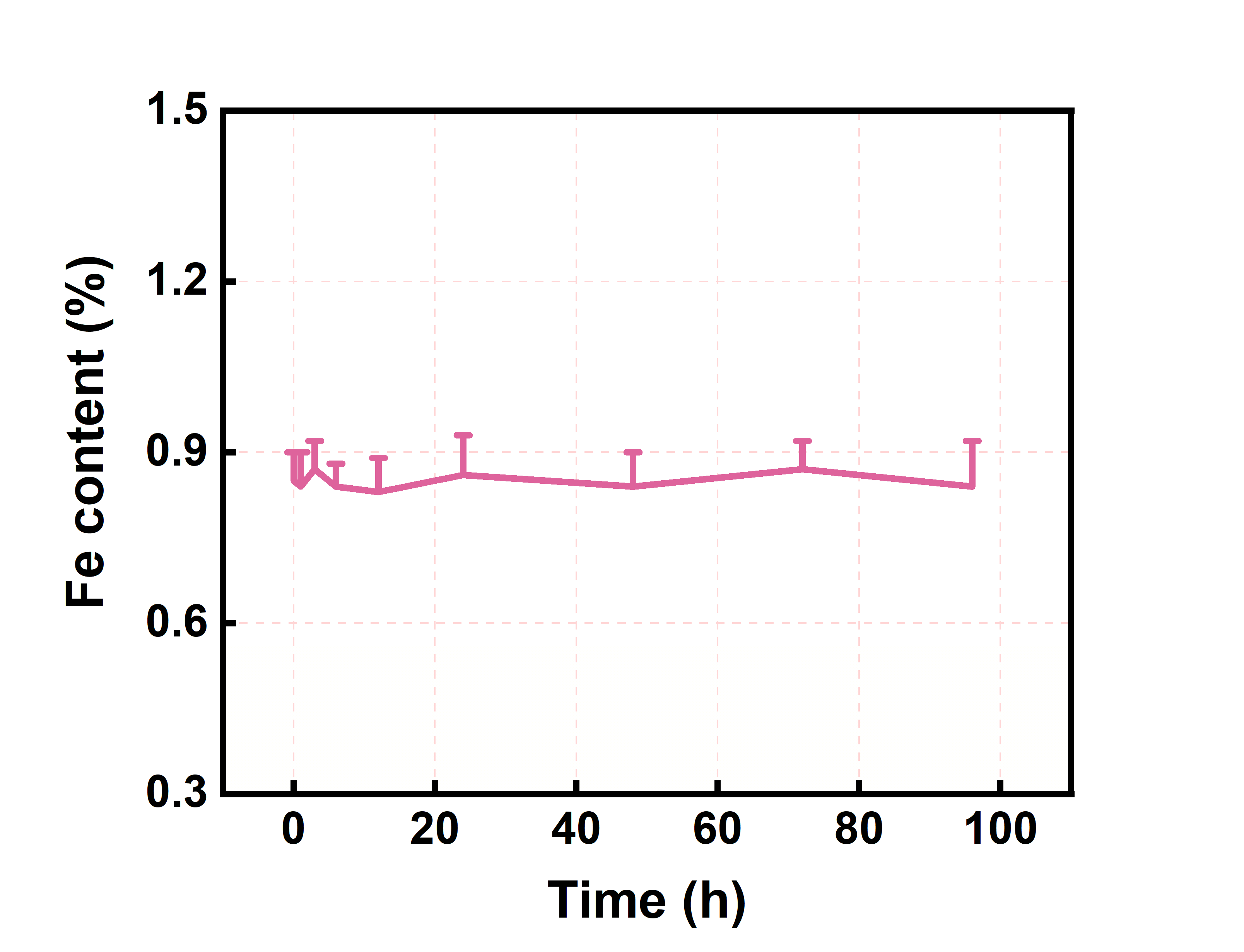


**Figure S6.** Temporal profile of Fe content in blood co-incubated with Fe-SAE@D. Data are presented as mean ± standard deviation (n=3).


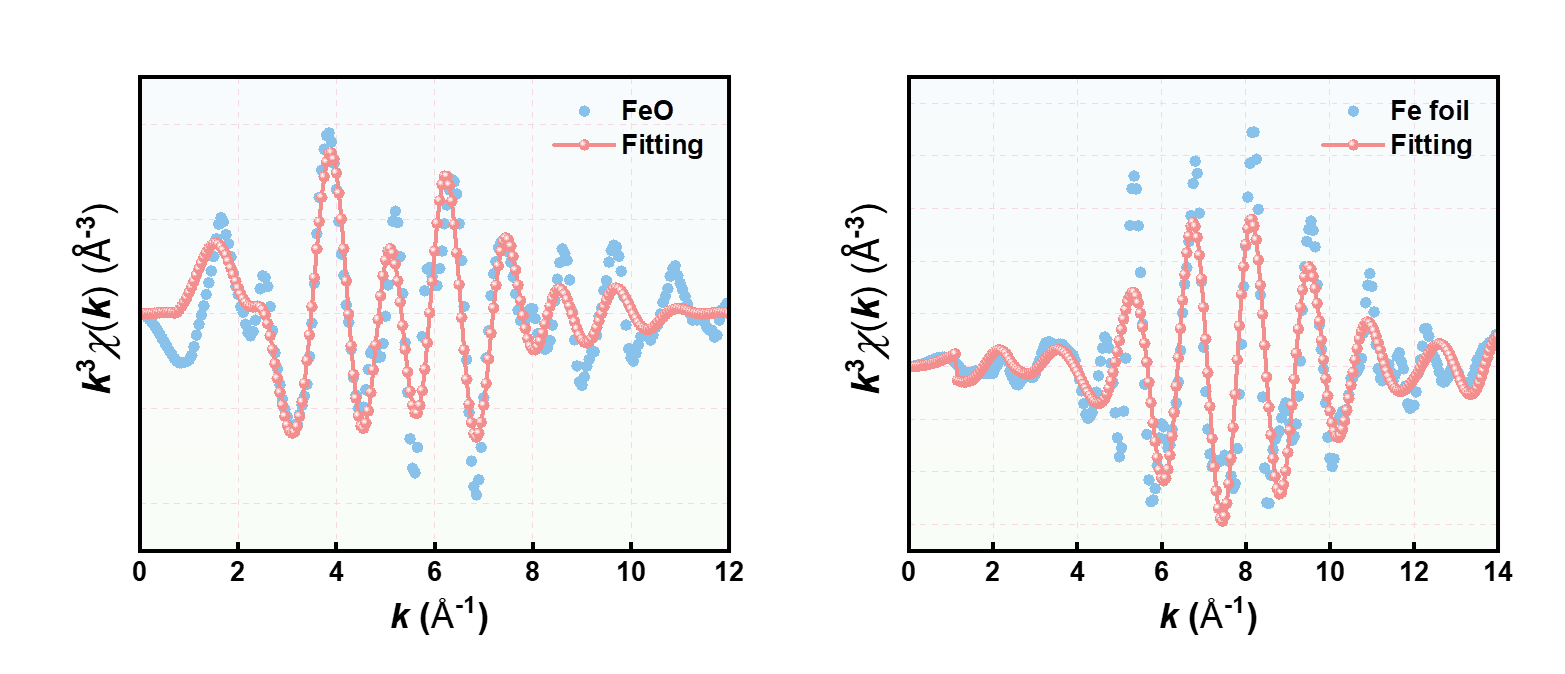


**Figure S7**. EXAFS fitting curves of FeO and Fe Foil at the k space.

**Table S1.** EXAFS data fitting results of Samples.

| Sample | Path | *CN^a^* | *R*(Å)*^b^* | *σ*^2^ (Å^2^)*^c^* | Δ*E*_0_(eV)*^d^* | *R* factor |
| --- | --- | --- | --- | --- | --- | --- |
| Fe K-edge (*Ѕ*_0_^2^=0.767) | | | | | | |
| Fe foil | Fe-Fe | 8.0* | 2.456±0.008 | 0.0030 | 4.6 | 0.0050 |
|  | Fe-Fe | 6.0* | 2.849±0.010 | 0.0031 |  |  |
| FeO | Fe-O | 6.2±0.8 | 2.094±0.013 | 0.0040 | 3.4 | 0.0134 |
|  | Fe-Fe | 11.9±0.8 | 3.081±0.014 | 0.0158 |  |  |
| FePc | Fe-N | 3.5±0.4 | 1.940±0.007 | 0.0050 | 9.8 | 0.0188 |
|  | Fe-N-C | 4.8±1.2 | 2.962±0.012 | 0.0032 |  |  |
| Fe-SAE | Fe-N | 3.7±0.4 | 1.961±0.026 | 0.0141 | -4.9 | 0.0113 |

*^a^CN*, coordination number; *^b^R*, the distance between absorber and backscatter atoms; *^c^σ*^2^, the Debye Waller factor value; *^d^ΔE*_0_, inner potential correction to account for the difference in the inner potential between the sample and the reference compound; *R* factor indicates the goodness of the fit. *S*0^2^ was fixed to 0.767, according to the experimental EXAFS fit of Fe foil by fixing *CN* as the known crystallographic value. * This value was fixed during EXAFS fitting, based on the known structure of Fe. Fitting conditions: *k* range：3.0 - 9.0; *R* range: 1.2-2.5; fitting space: R space; *k*-weight = 2. A reasonable range of EXAFS fitting parameters: 0.800 < *Ѕ*_0_^2^ < 1.000; *CN >* 0; *σ*^2^ > 0 Å^2^; |Δ*E*_0_| < 15 eV; *R* factor < 0.02.


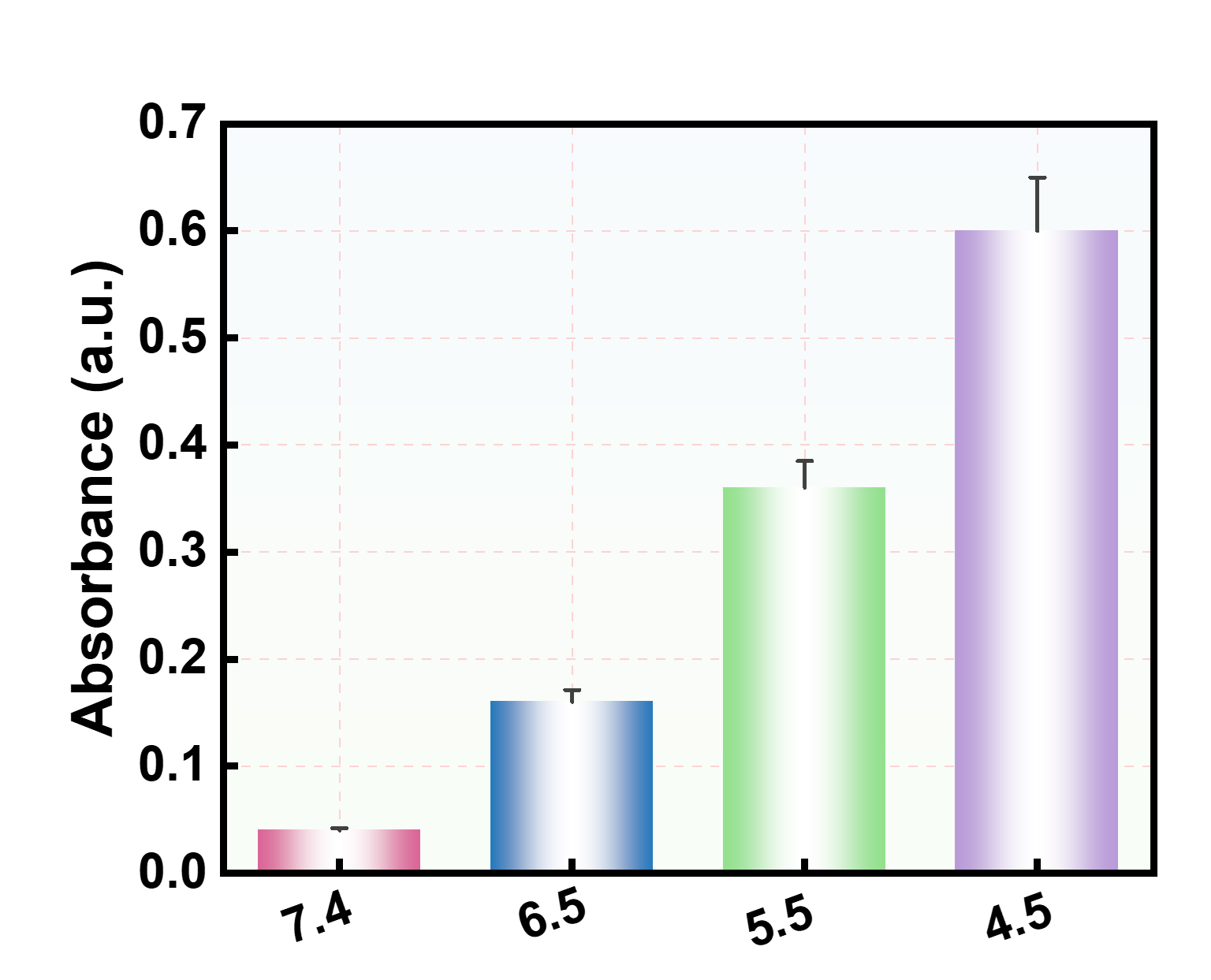


**Figure S8**. TMB assay for measuring POD-like activity of the Fe-SAE at the different pH.


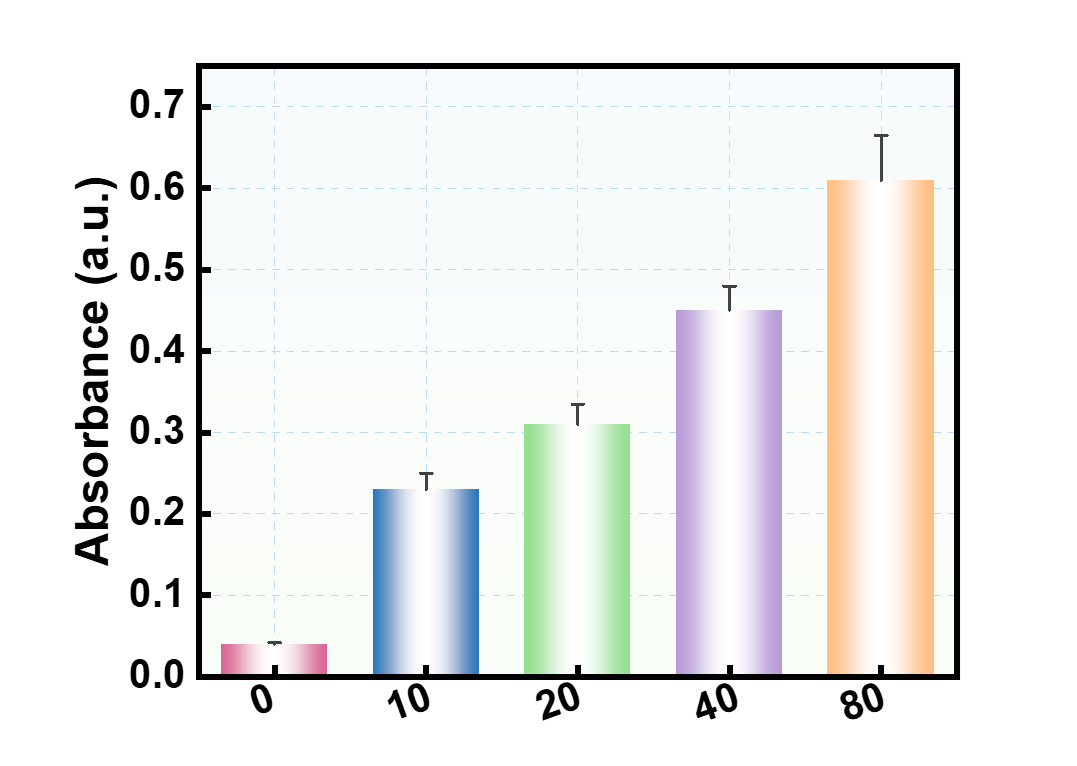


**Figure S9**. TMB assay for measuring POD-like activity after incubating the different concentrations of Fe-SAE.


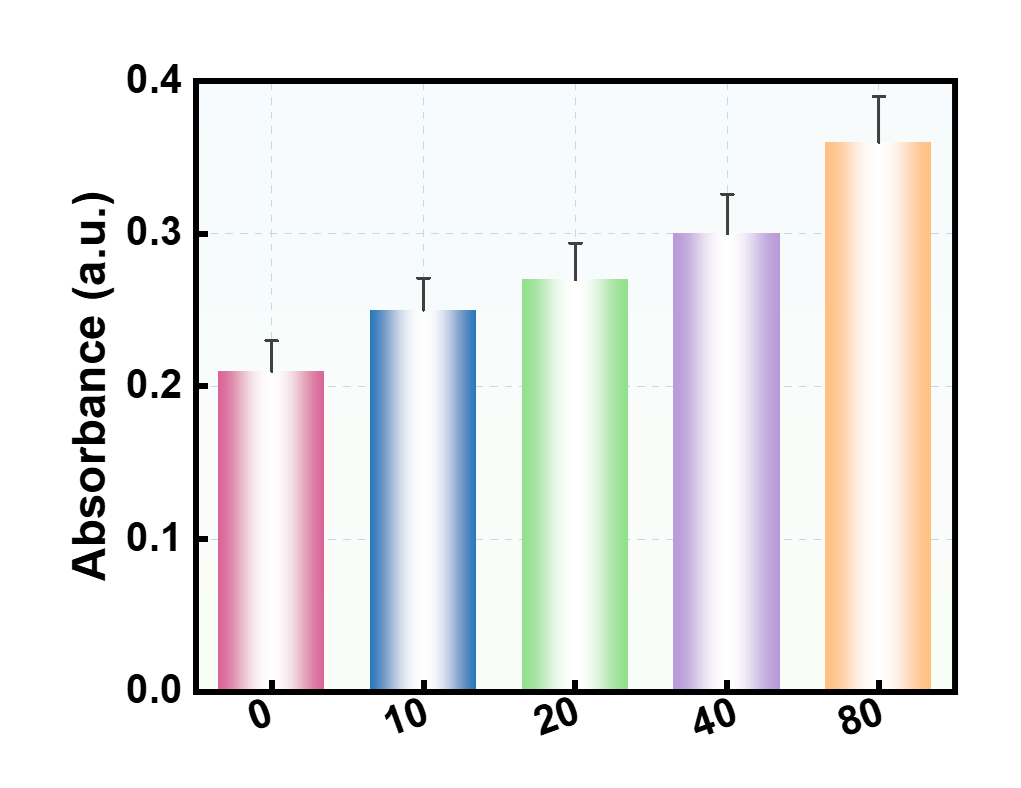


**Figure S10**. ABTS assay for measuring POD-like activity after incubating the different concentrations of Fe-SAE.


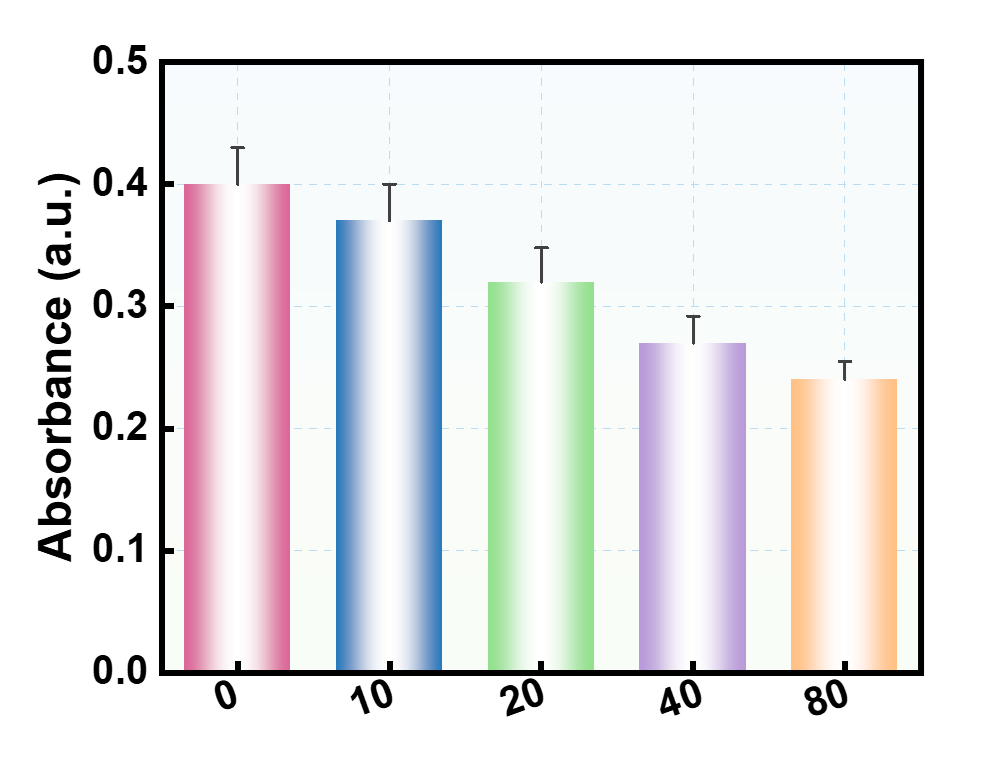


**Figure S11**. MB assay for measuring POD-like activity after incubating the different concentrations of Fe-SAE.


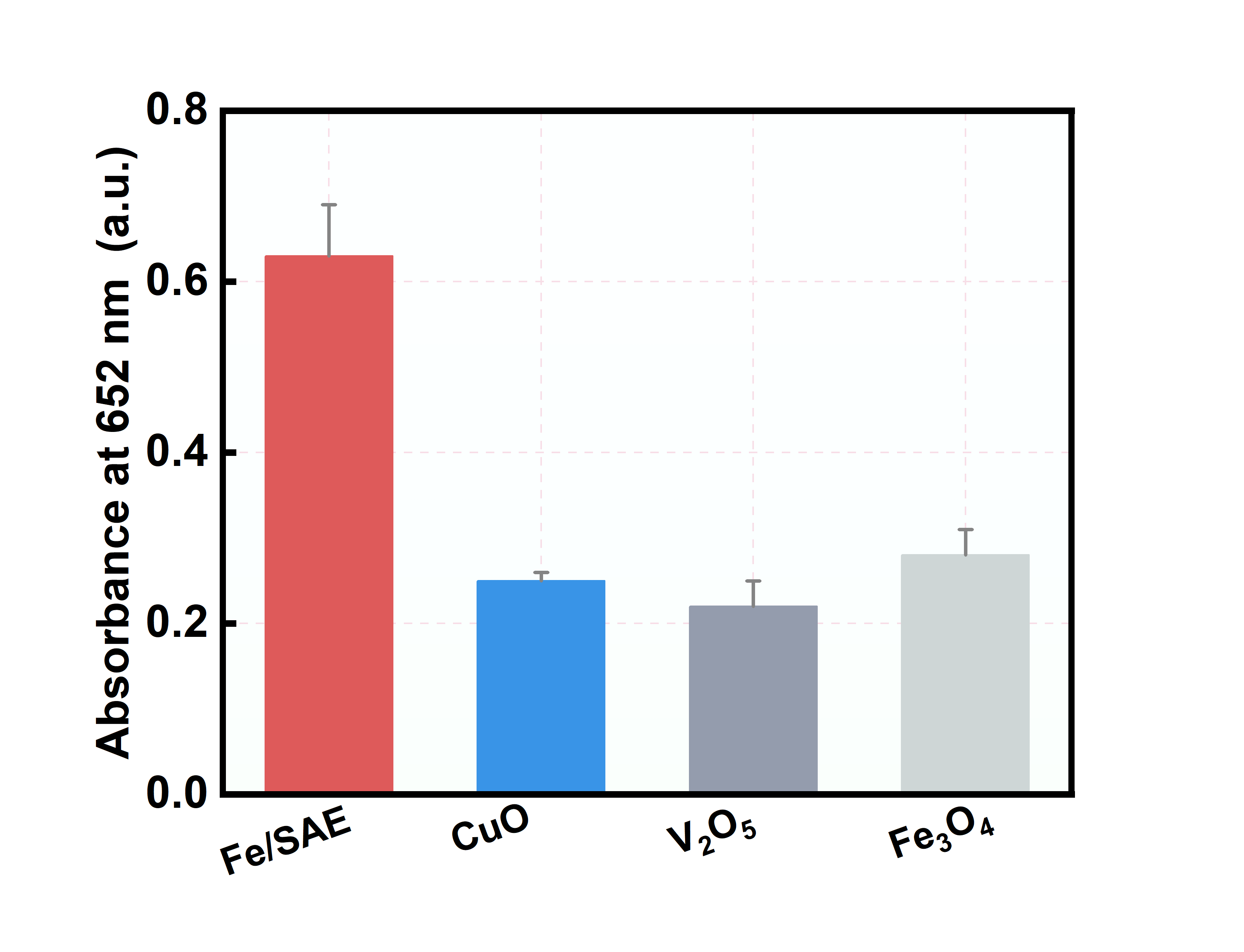


**Figure S12.** The POD-like activity of different nanozymes based on TMB assay.


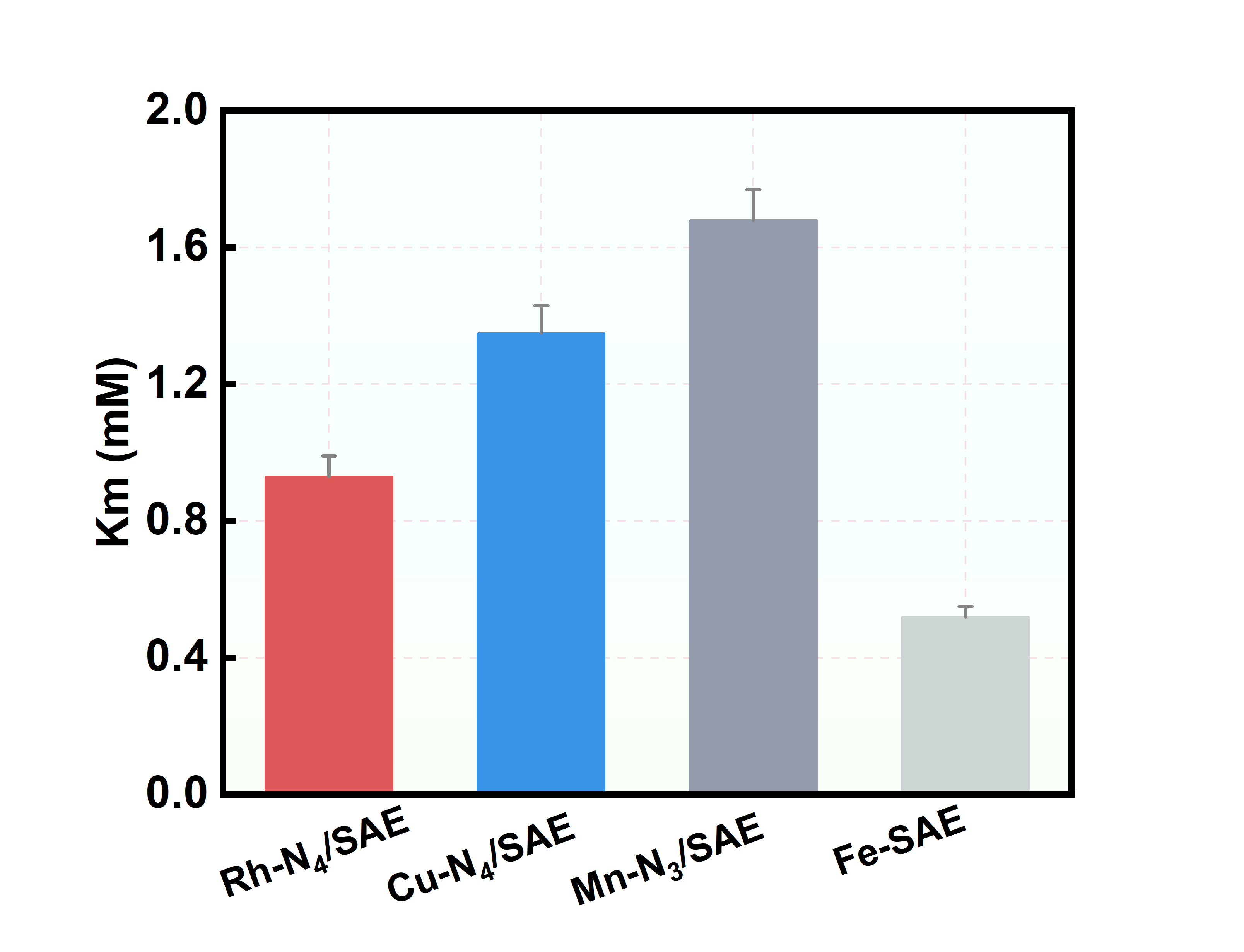


**Figure S13.** The Km values of different nanozymes.


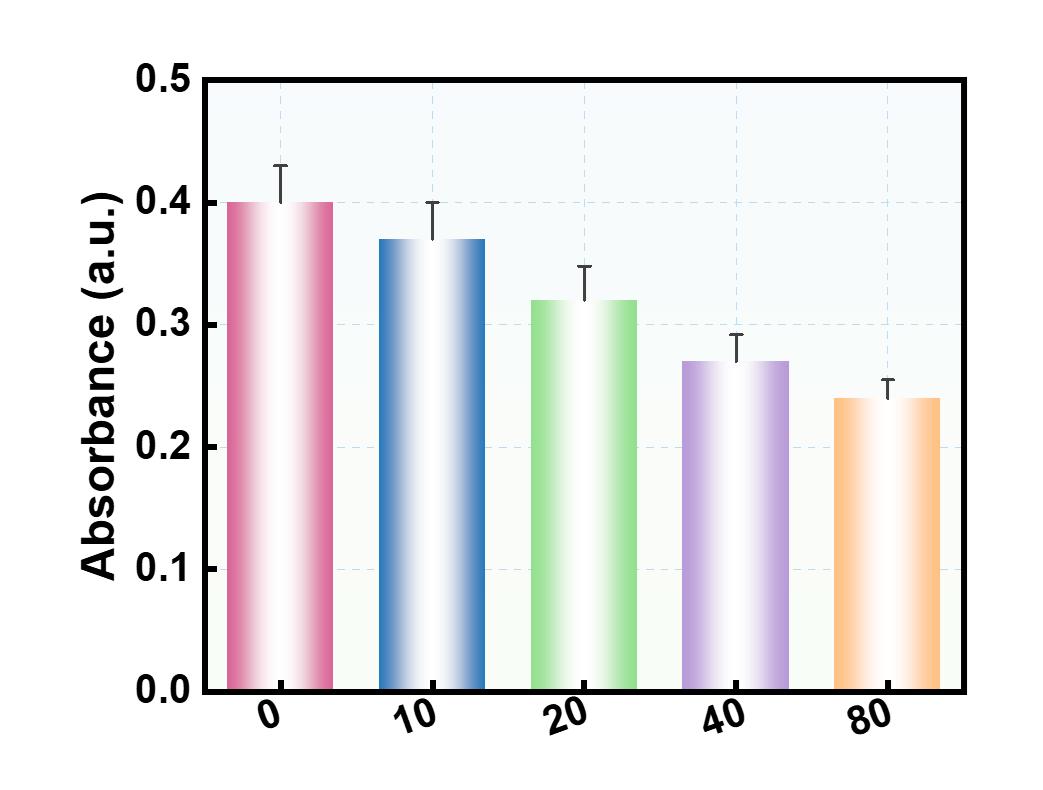


**Figure S14**. DTNB assay for measuring GSHOX-like activity after incubating the different concentrations of Fe-SAE.


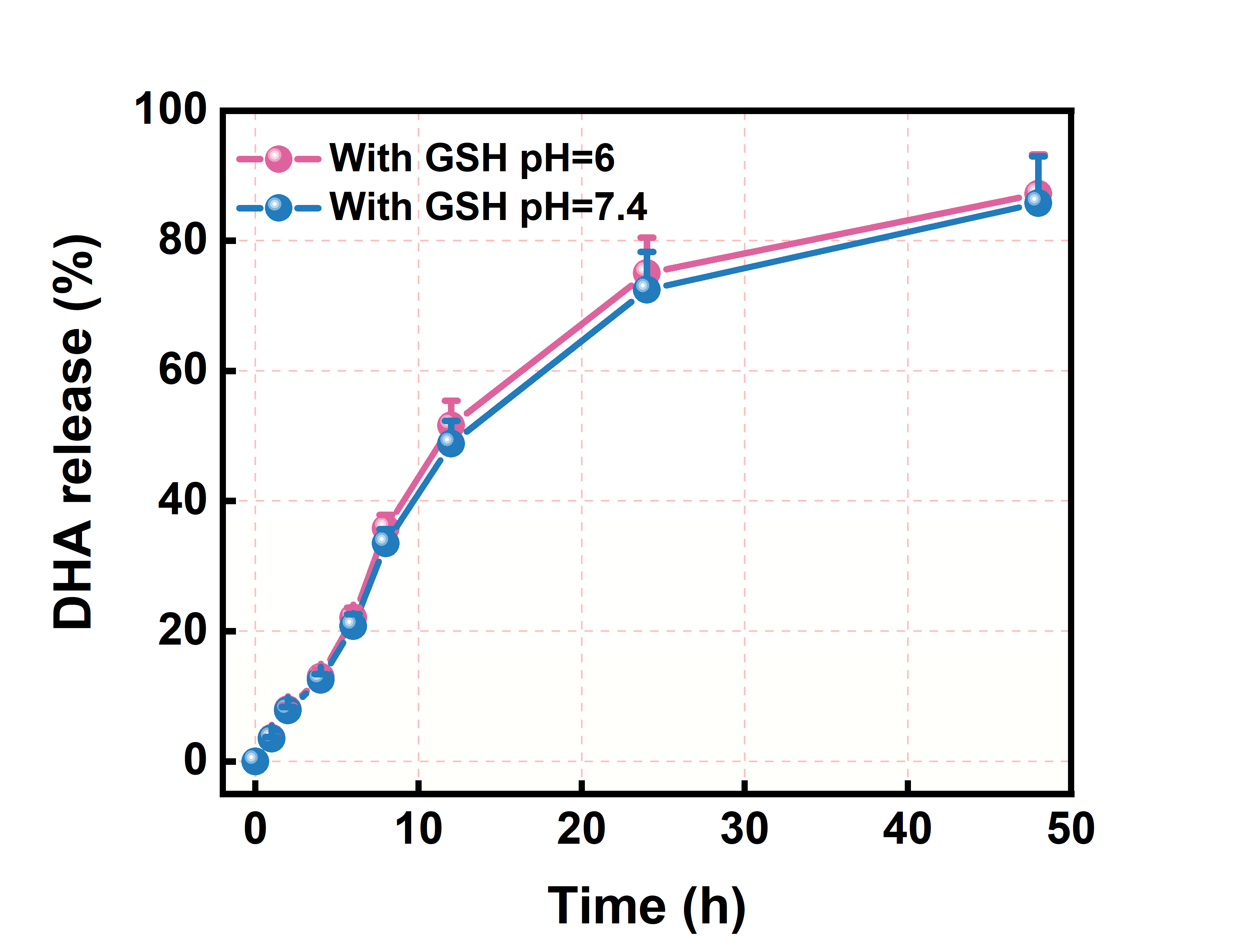


**Figure S15**. The DHA release curves in different pH conditions.


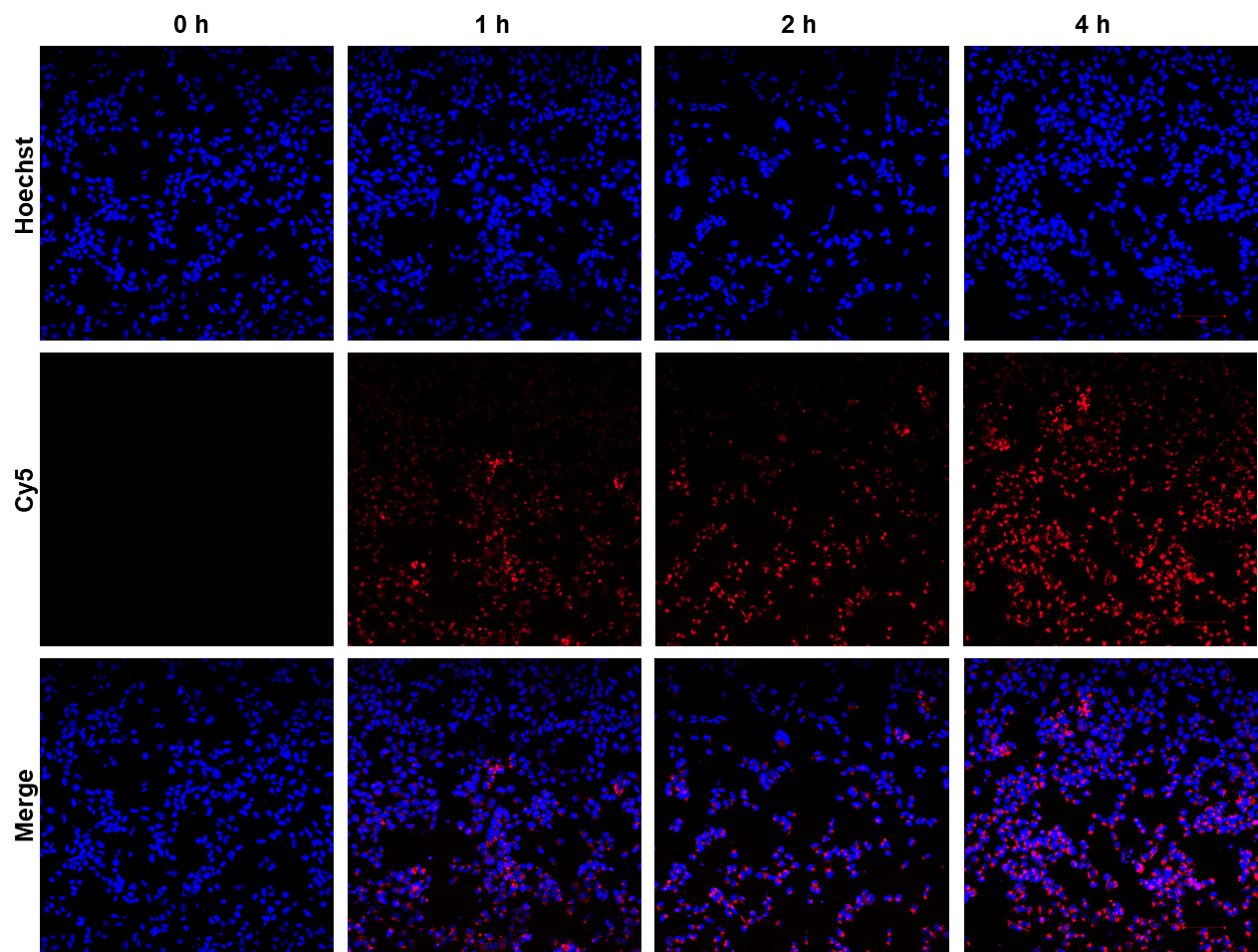


**Figure S16**. The CLSM images of tumor cells incubated with Cy5.5-labeled Fe-SAE@D.


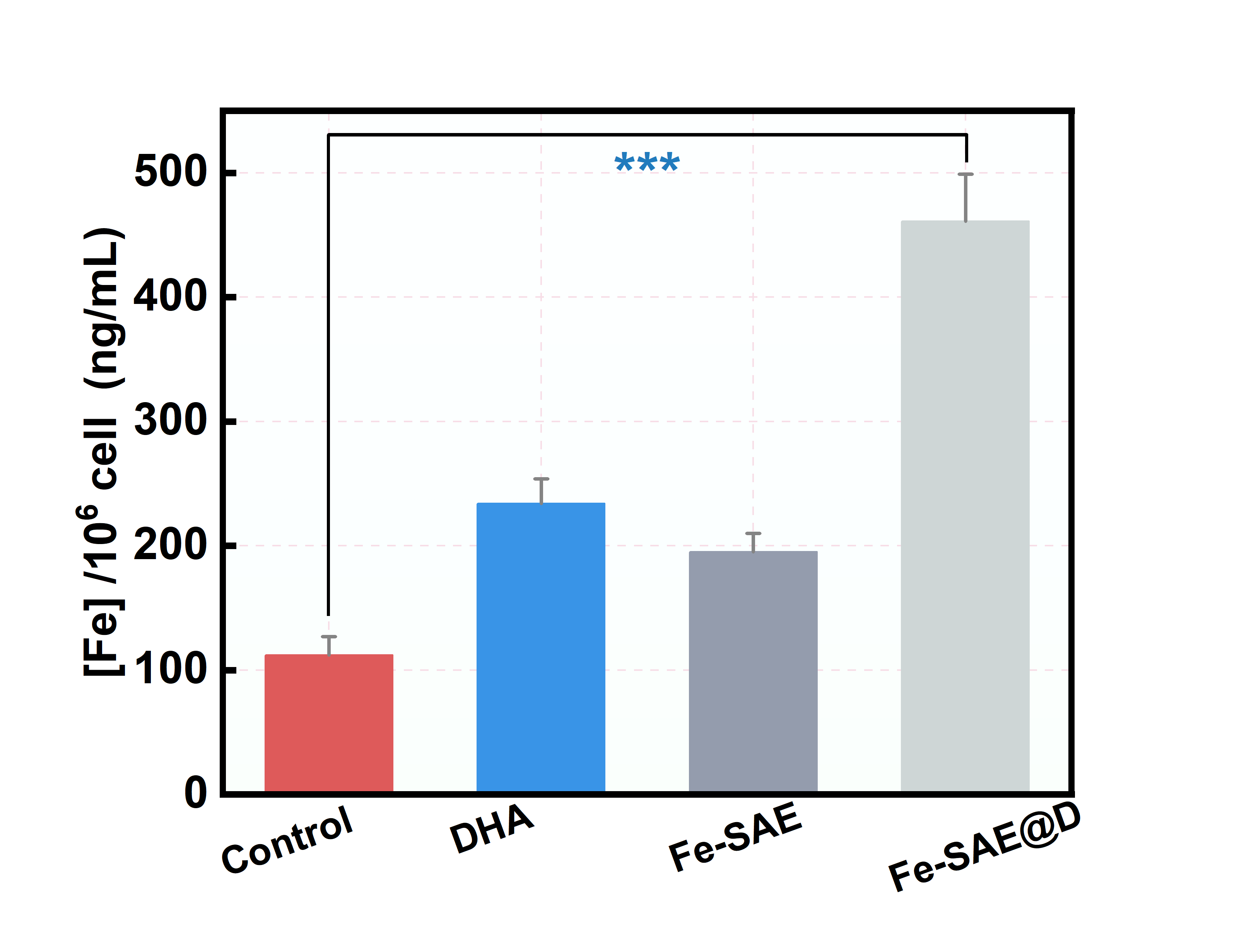


**Figure S17**. Intracellular Fe content in GL261 cells after 24 hours of treatment with different formulations, *** p < 0.001.


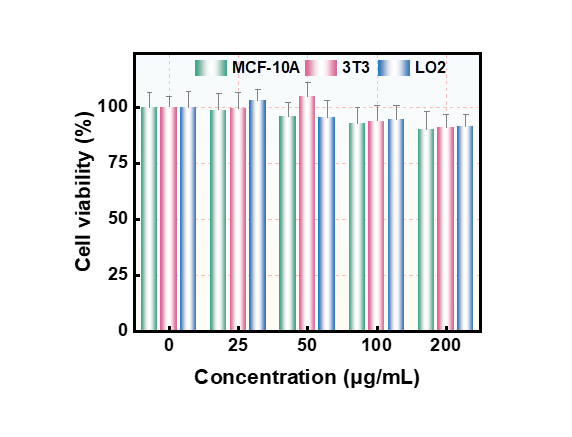


**Figure S18**. The CCK-8 results of different cells.


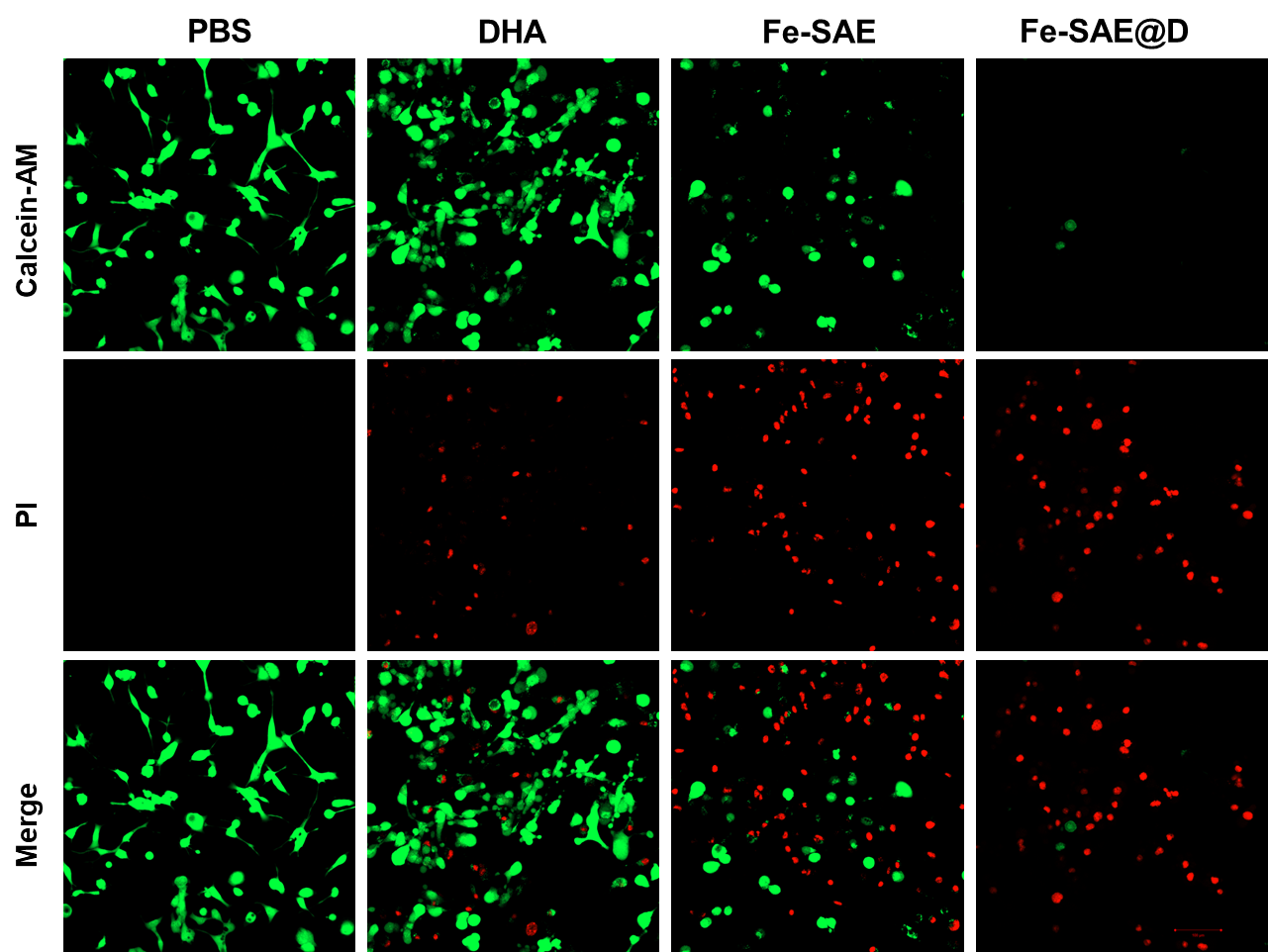


**Figure S19**. Calcein-AM/PI co-stained GL261 cells incubated with various formulations.


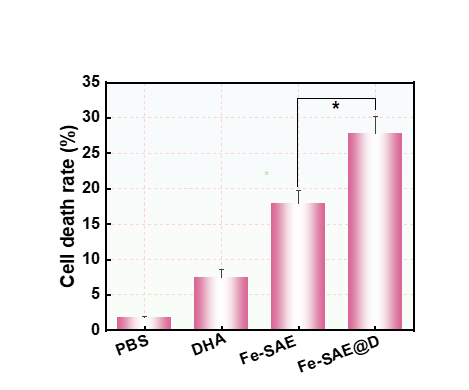


**Figure S20**. Flow cytometry measurements of GL261 cells after incubation with varying formulations.


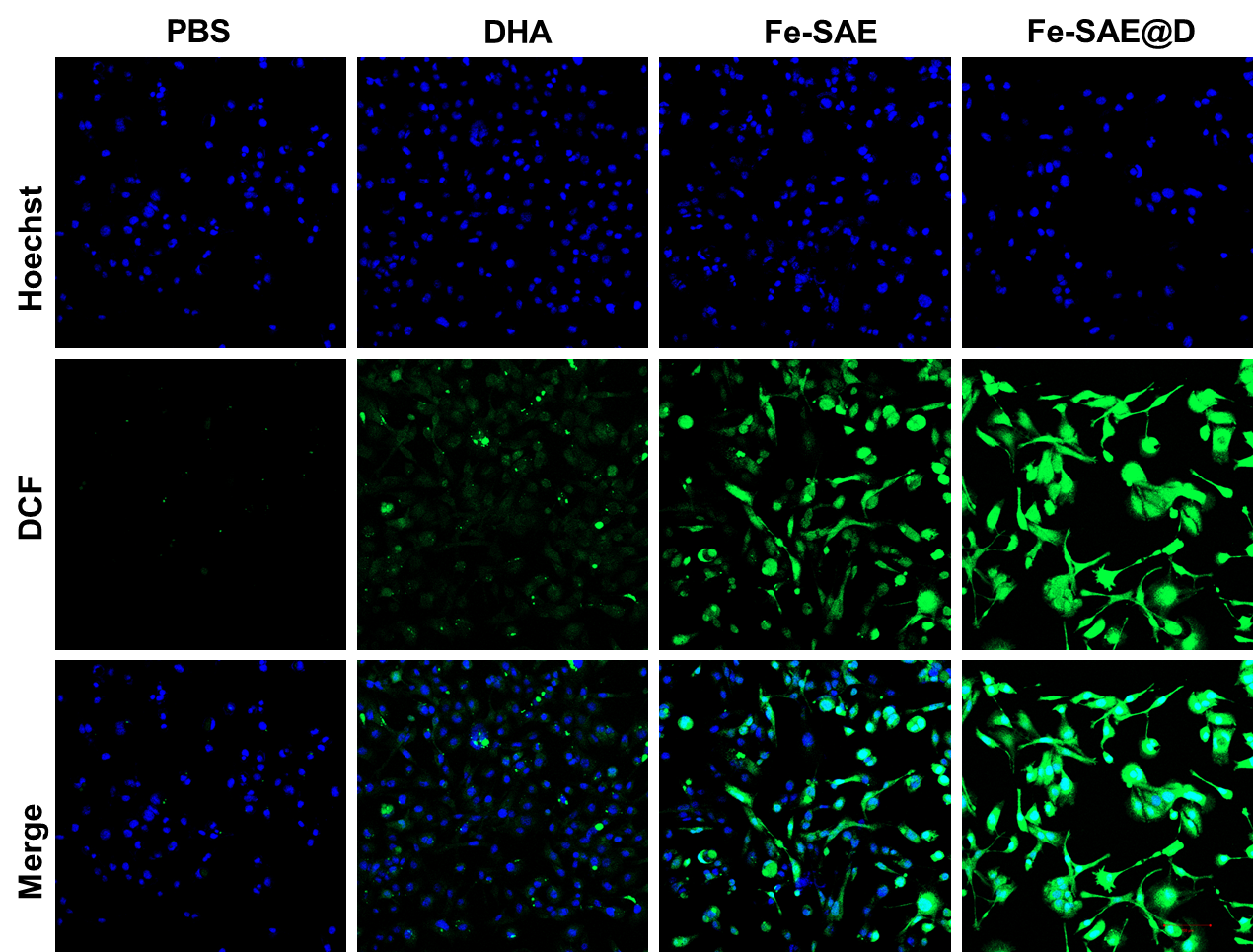


**Figure S21**. CLSM images of DCF in GL261 cells treated with different formulations.


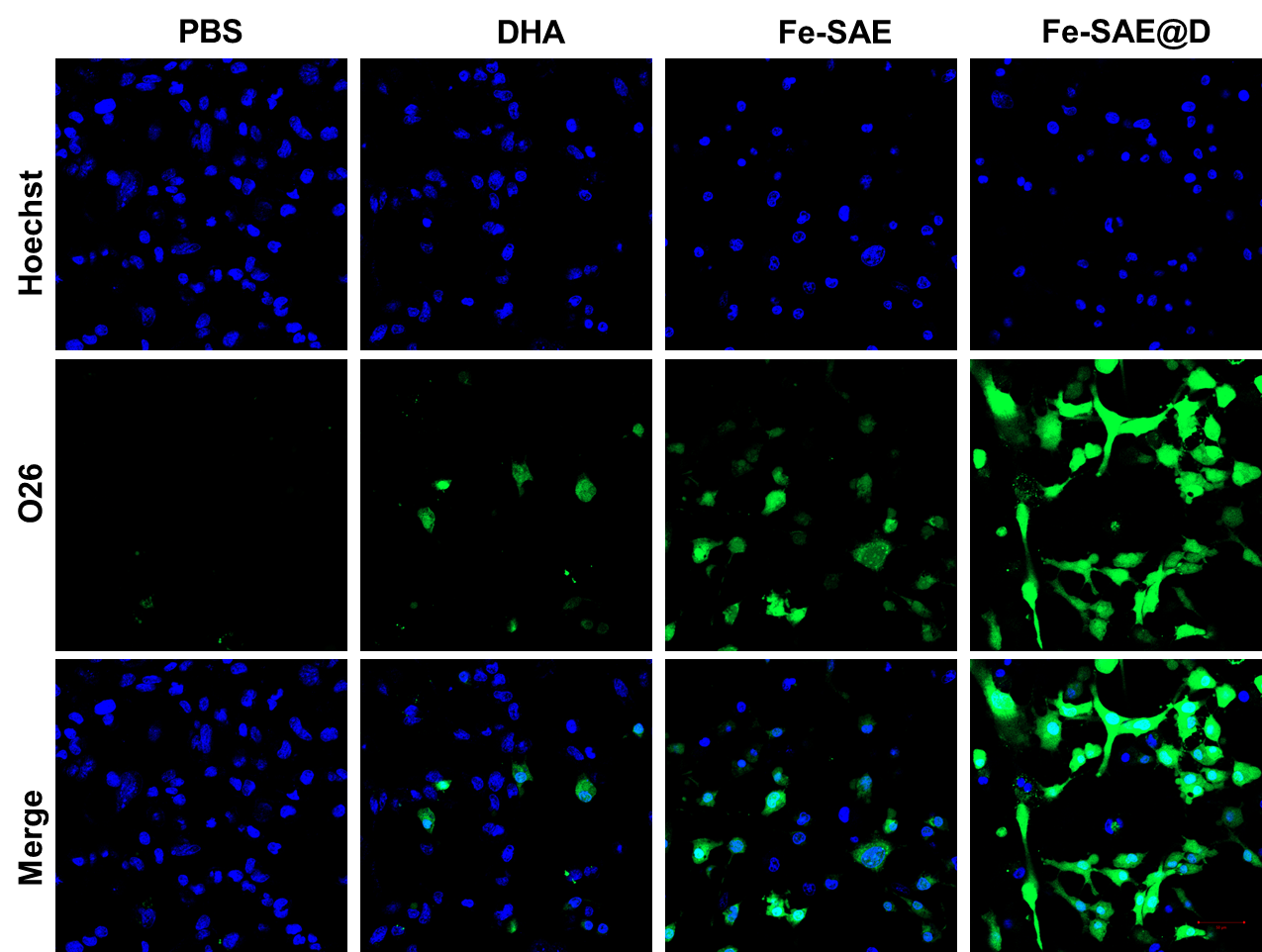


**Figure S22**. CLSM images of H_2_S in GL261 cells treated with different formulations.


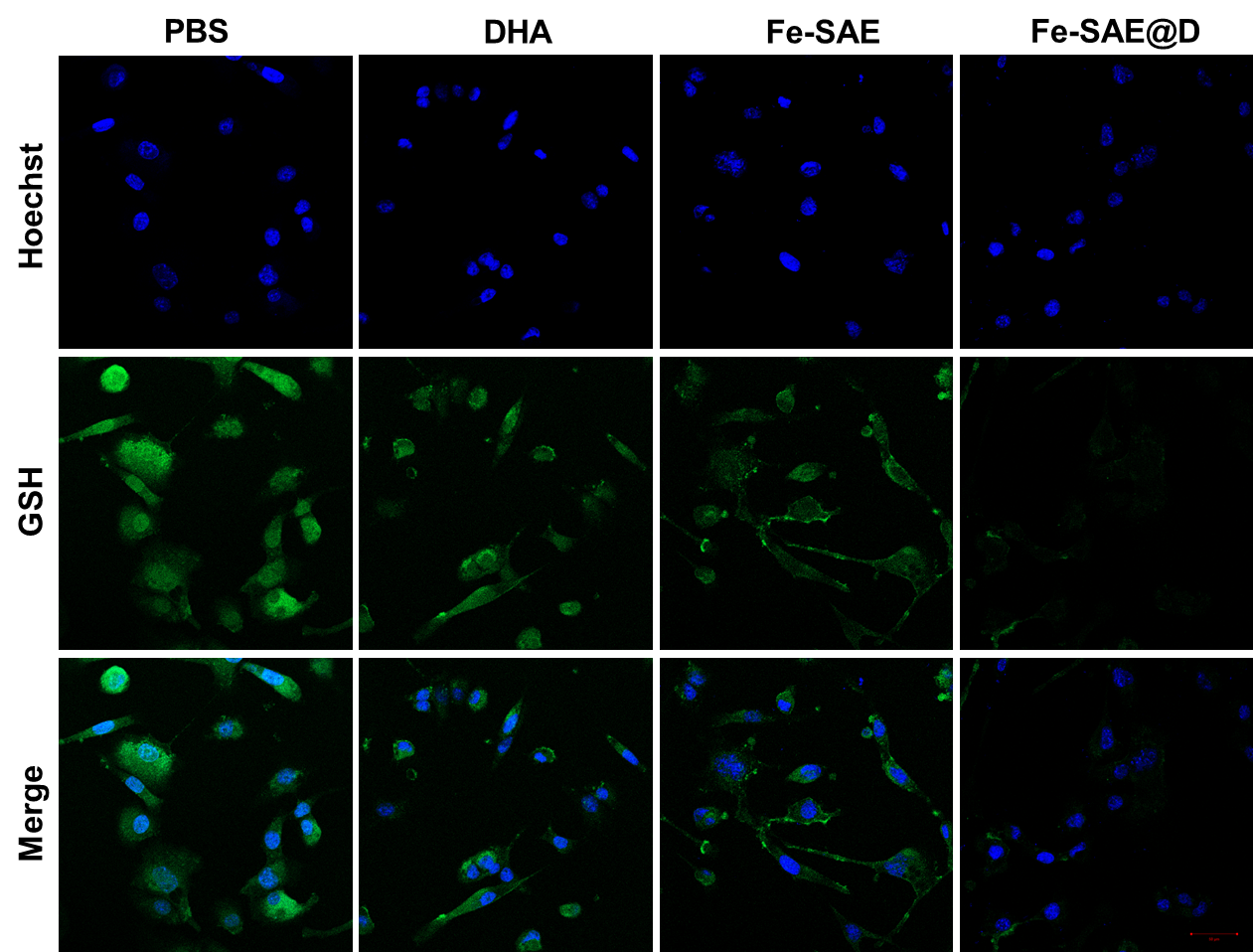


**Figure S23**. CLSM images of GSH in GL261 cells treated with different formulations.


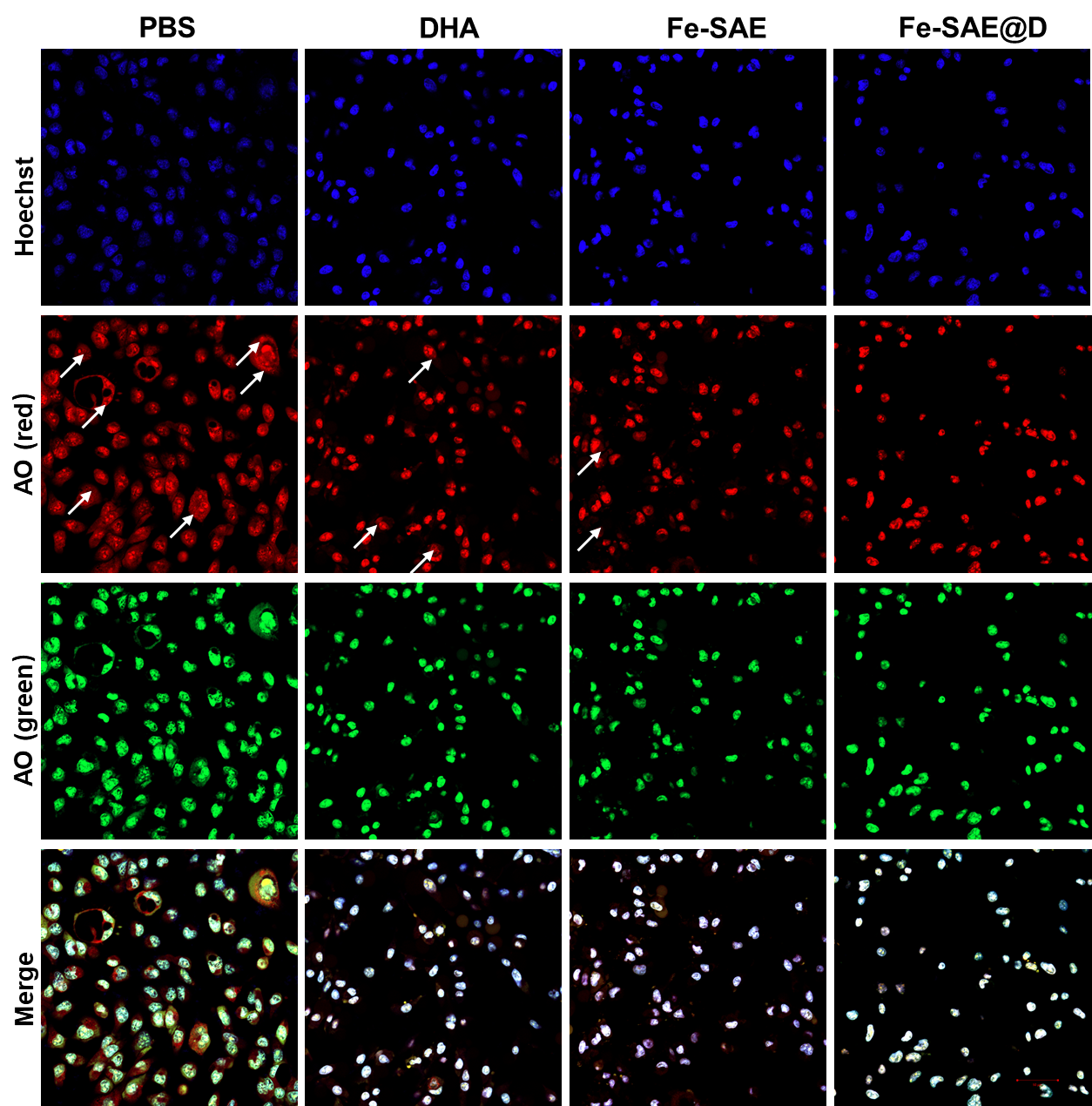


**Figure S24**. CLSM images of AO-stained GL261 cells after 24 h of incubation with different formulations.


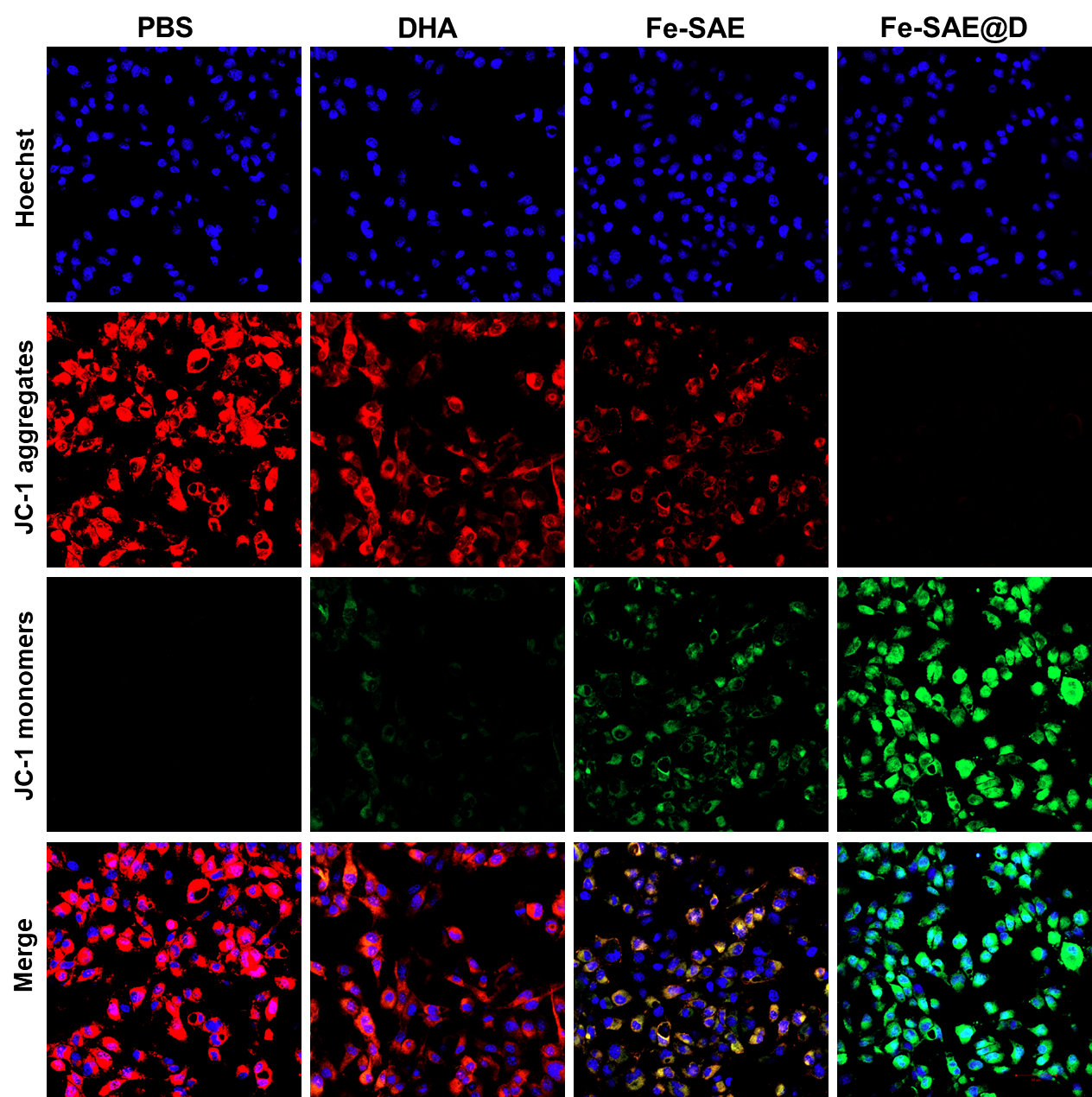


**Figure S25**. JC-1 fluorescence images of tumor cells following different treatments.


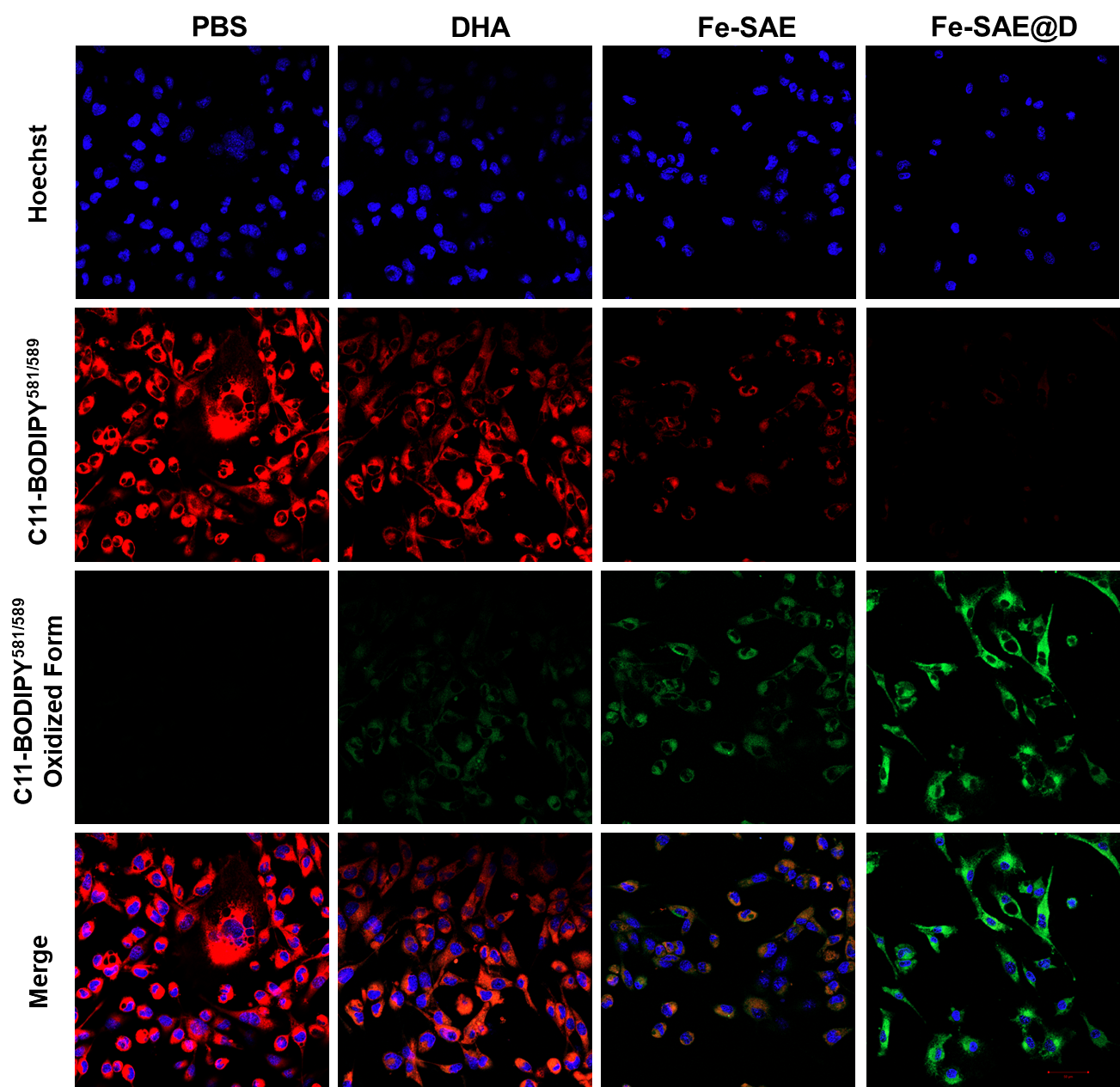


**Figure S26**. The confocal images of fluorescent probe C11-BODIPY581/589-stained cancer cells following various treatments.


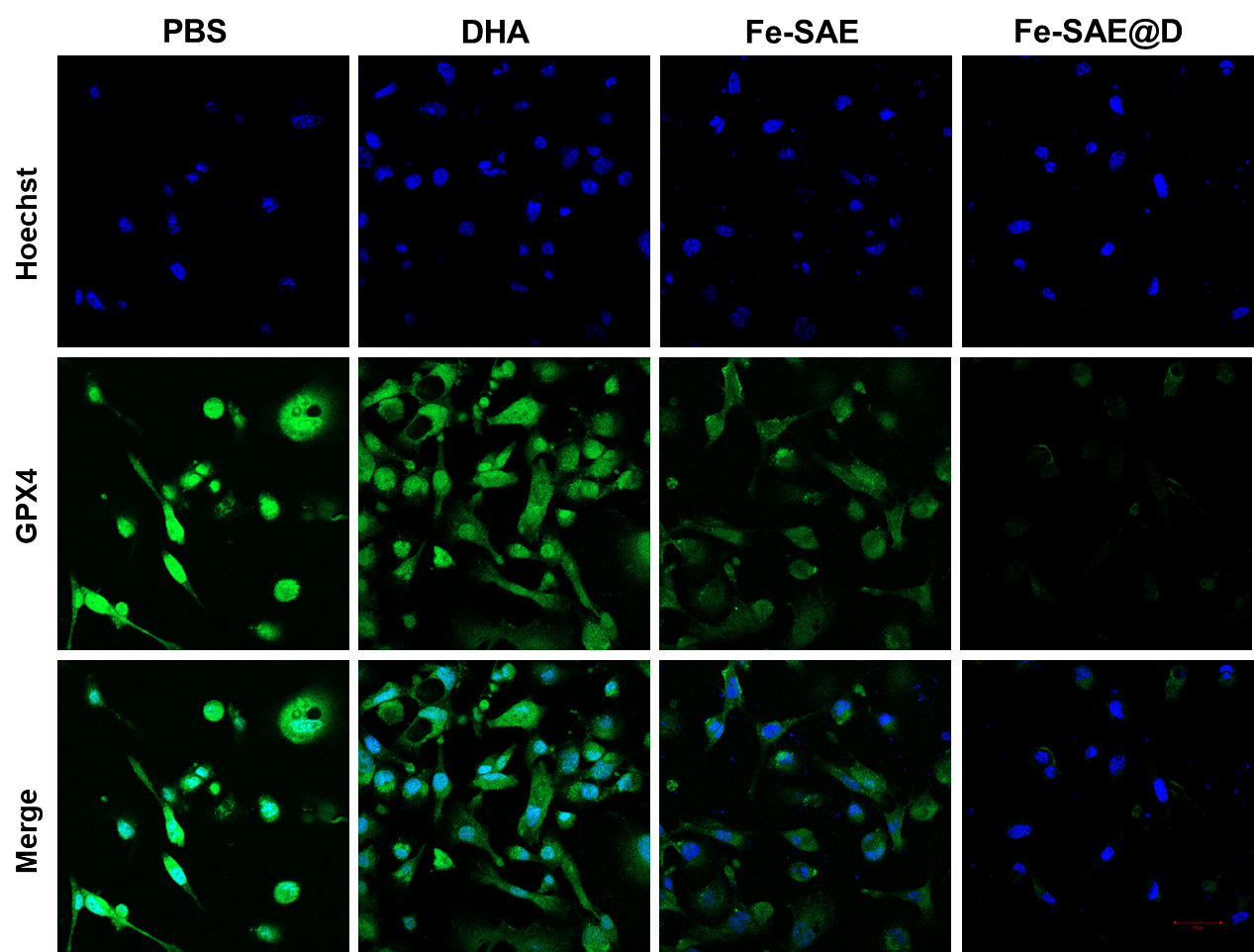


**Figure S27.** CLSM images of GPX4 expression in GL261 cells treated with different formulations.


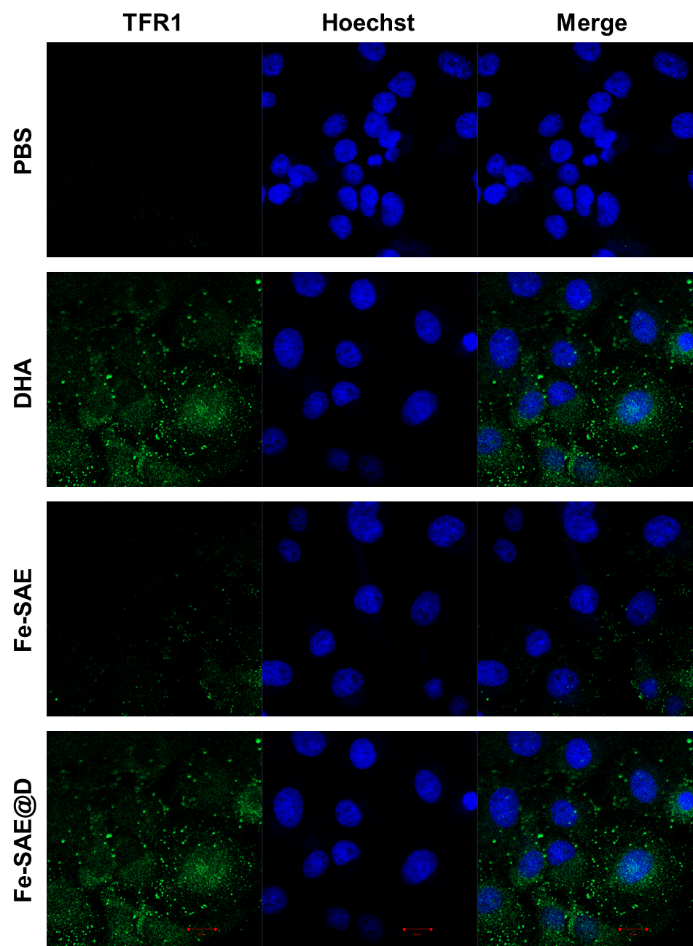


**Figure S28.** The expression of TFR in tumor cells exposed to various formulations.


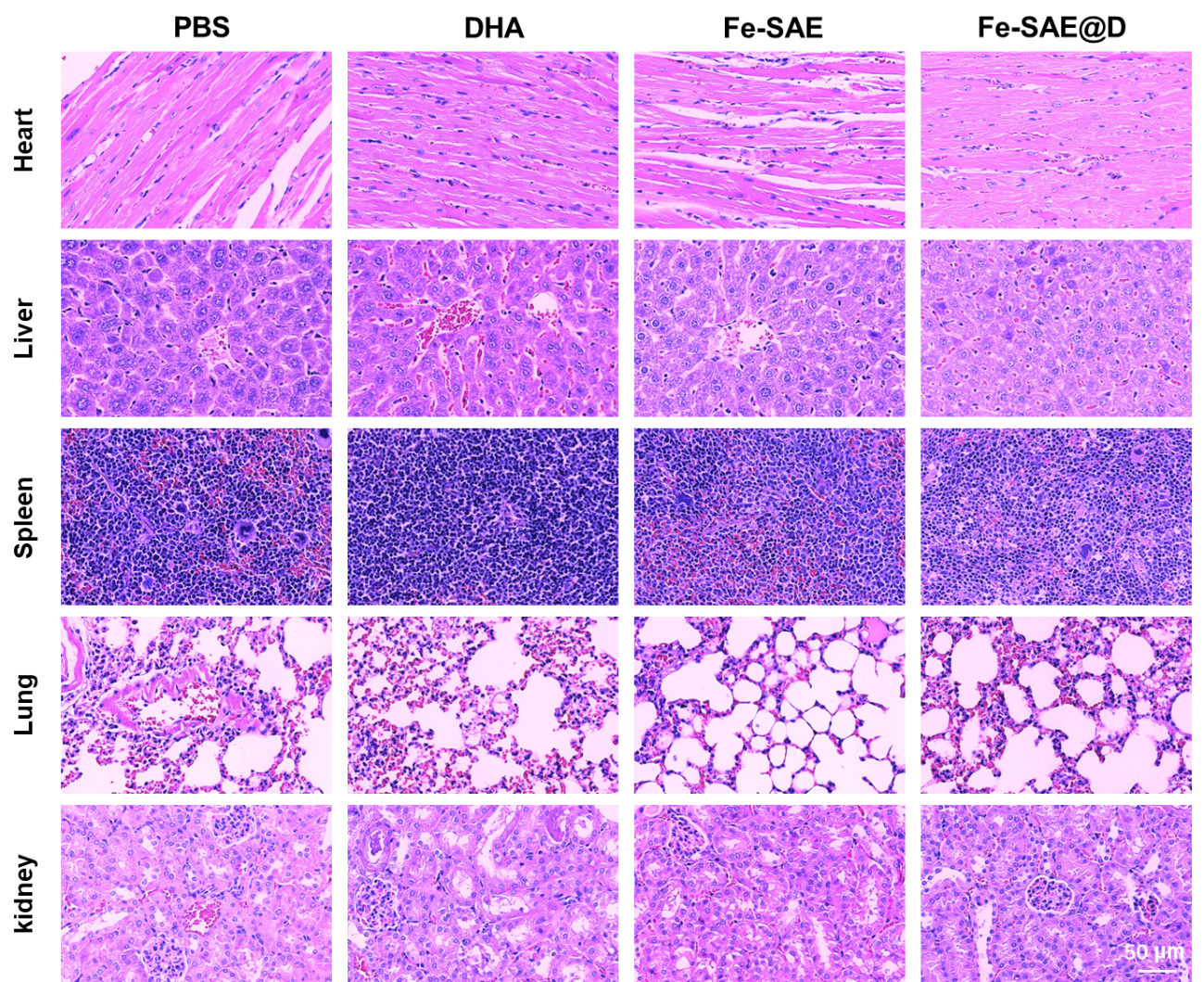


**Figure S29**. H&E staining of the major organs.


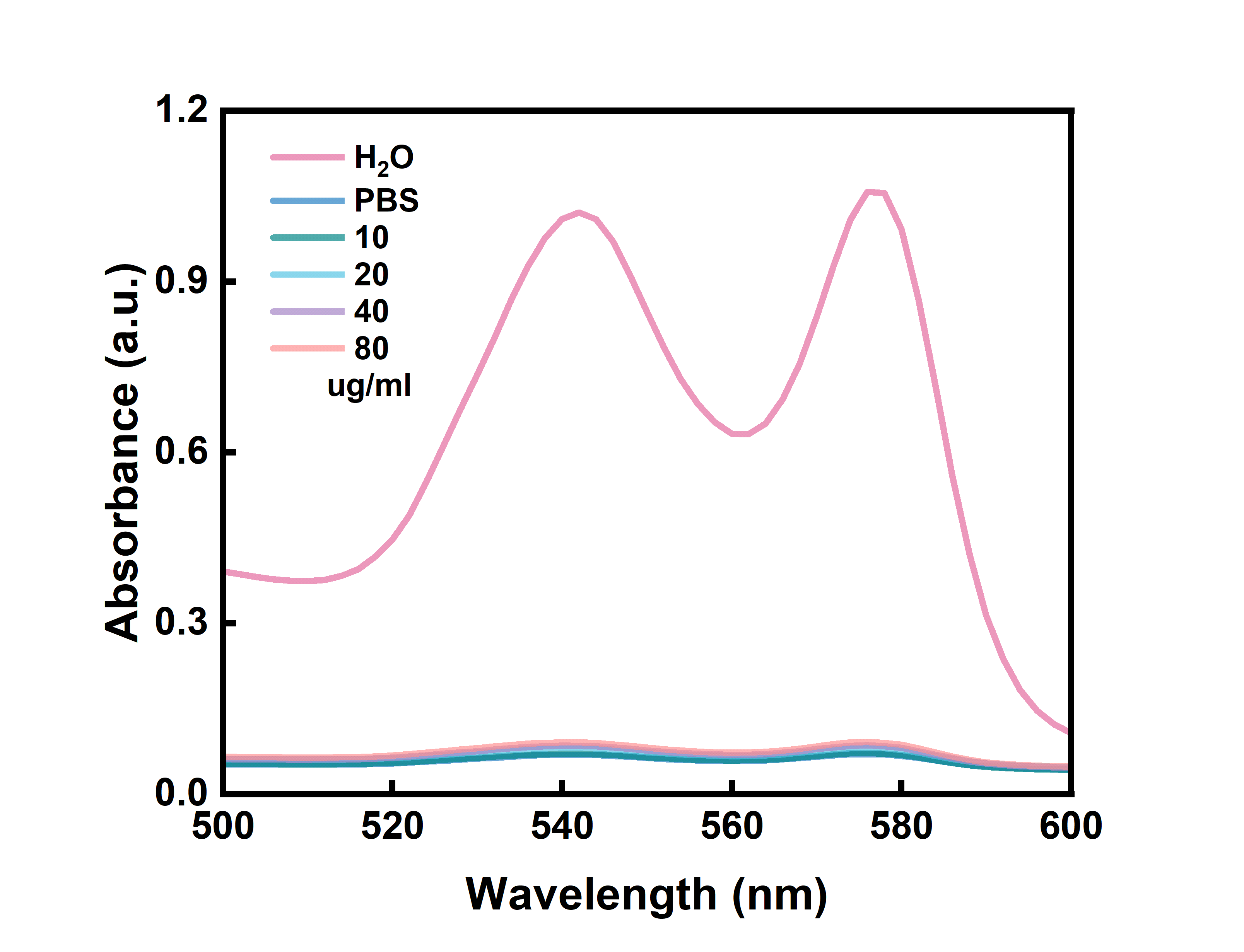

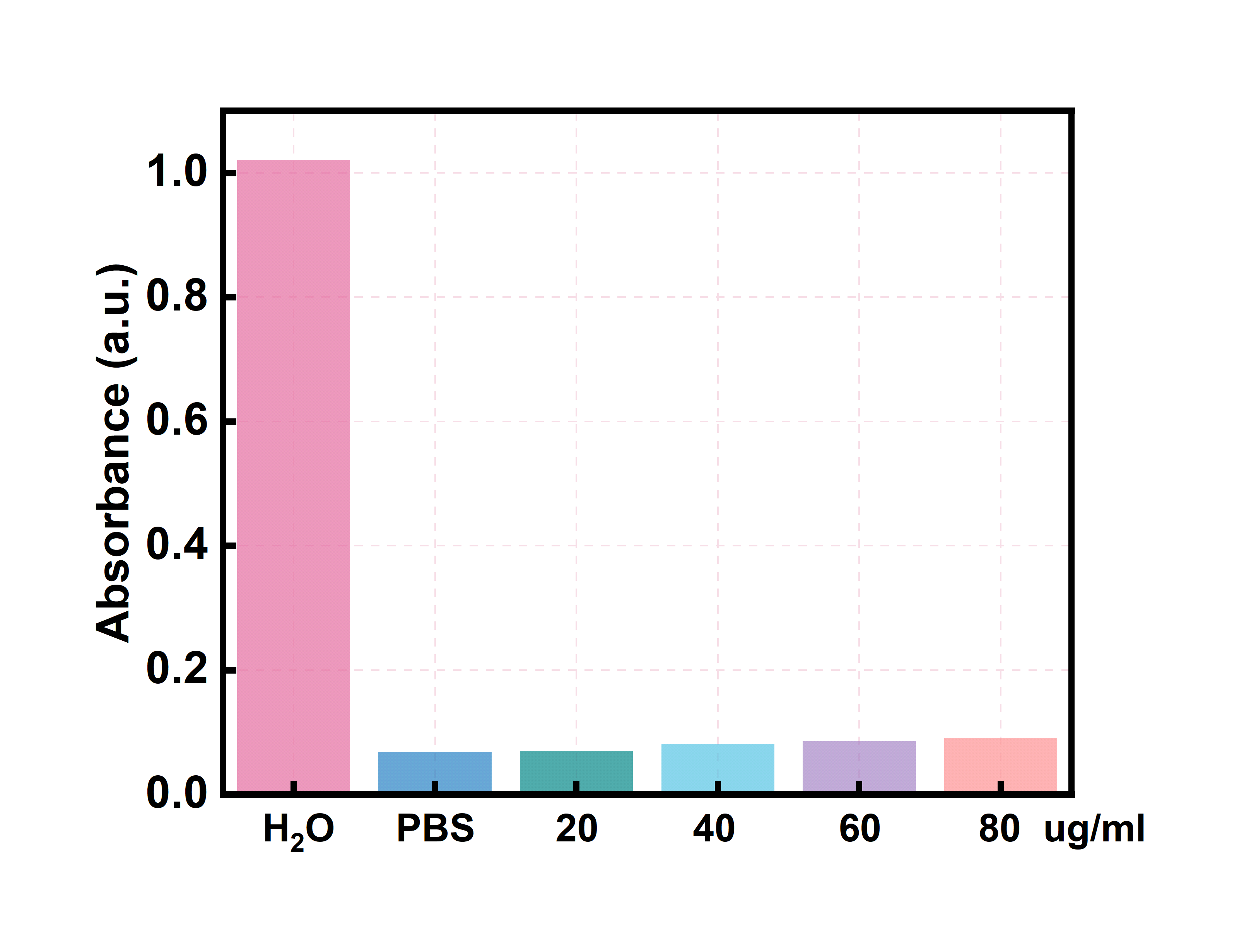


**Figure S30.** Ultraviolet-visible spectra and hemolysis rates of red blood cell solutions measured after being placed in deionized water and PBS solutions with different concentrations of Fe-SAE@D NPs, respectively.


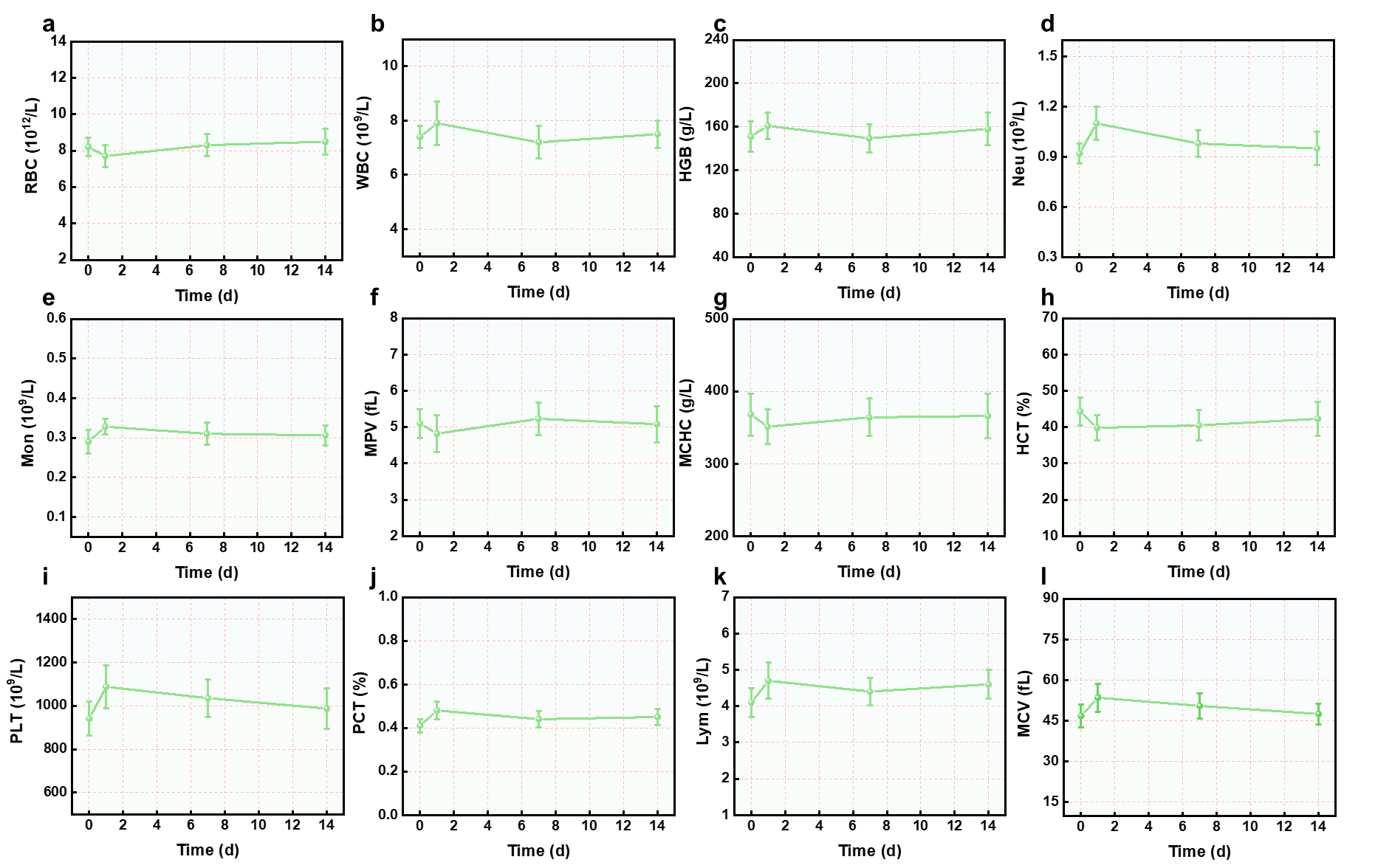
 **Figure S31.** Trend graphs of different routine blood test indices over time after mice were intravenously injected with Fe-SAE@D NPs via caudal vein. (a) RBC, Red Blood Cells. (b) WBC, White Blood Cells. (c) HGB, Hemoglobin. (d) Neu, Neutrophils. (e) Mon, Monocytes. (f) MPV, Mean Platelet Volume. (g) MCHC, Mean Corpuscular Hemoglobin Concentration. (h) HCT, Hematocrit. (i) PLT, Platelets. (j) PCT, Plateletcrit. (k) Lym, Lymphocytes. (l) MCV, Mean Corpuscular Volume.


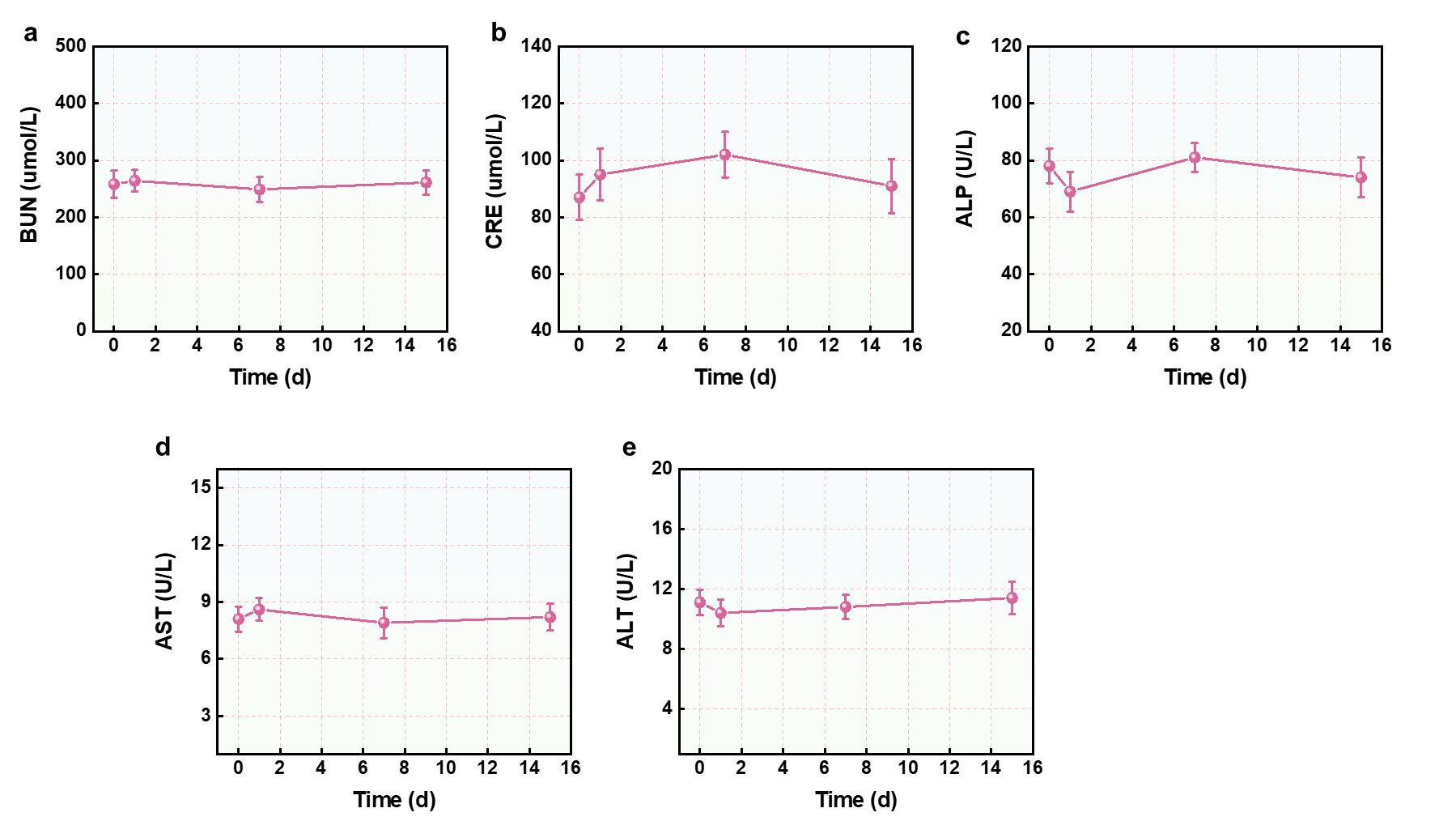


**Figure S32:** Trend graphs of various blood biochemical indices over time after Fe-SAE@D NPs were injected into mice via the caudal vein.


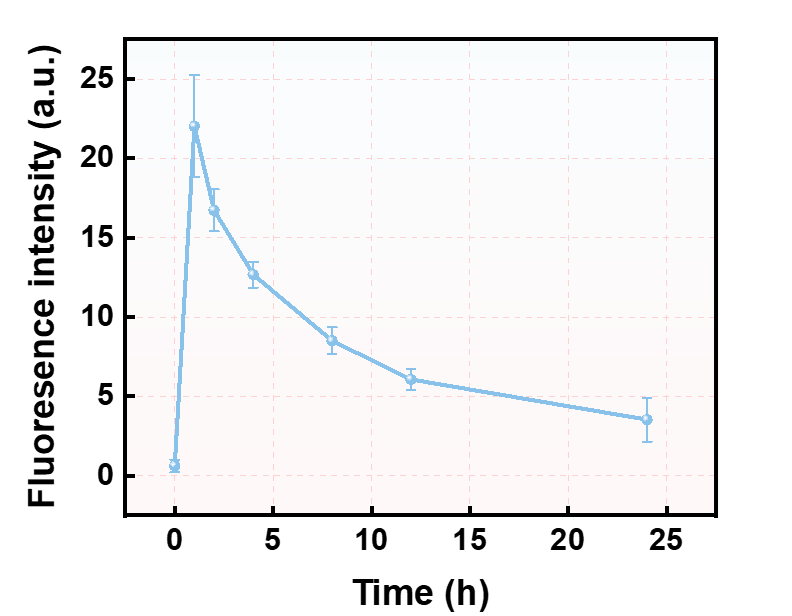


**Figure S33.** Time-dependent fluorescence intensity of Fe-SAE@D NPs at the tumor site in mice over 24 hours. Data are presented as mean ± standard deviation (n=3).


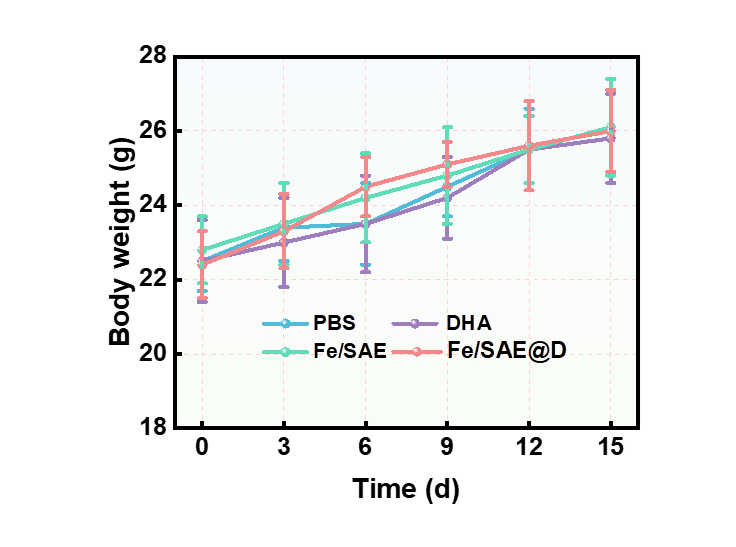


**Figure S34.** The body weight curves of mice following various treatments.


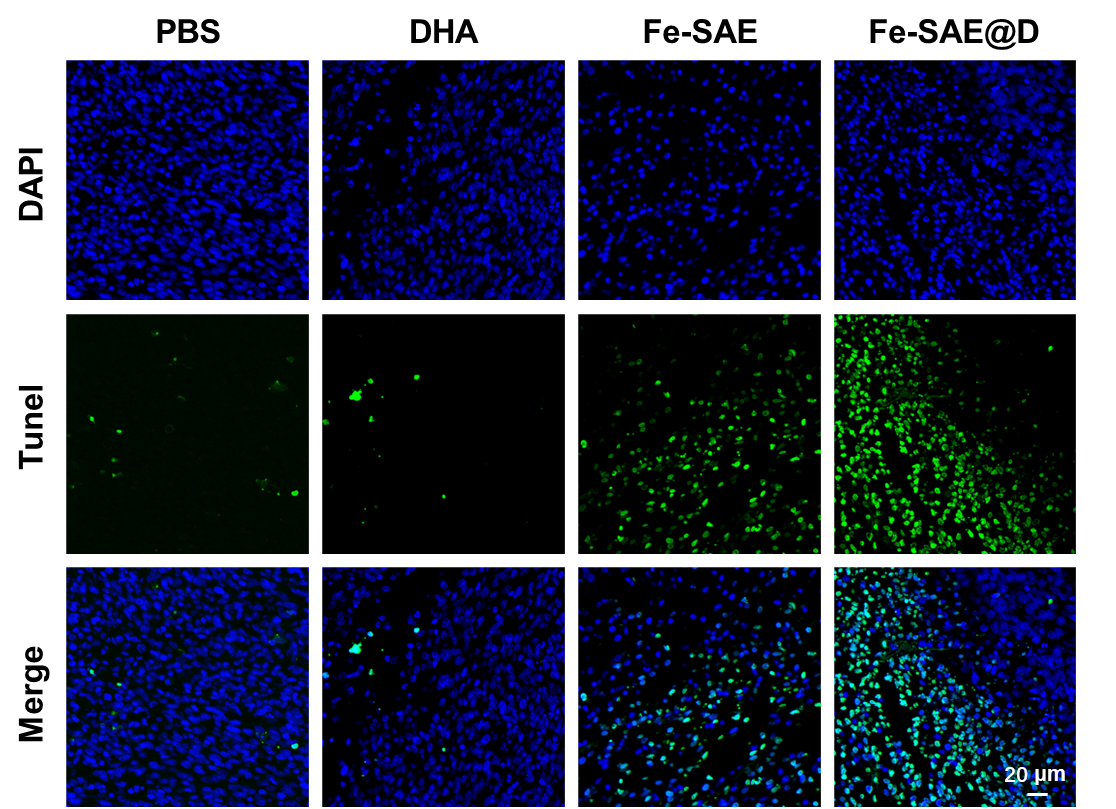


**Figure S35.** TUNEL staining of tumor tissue.


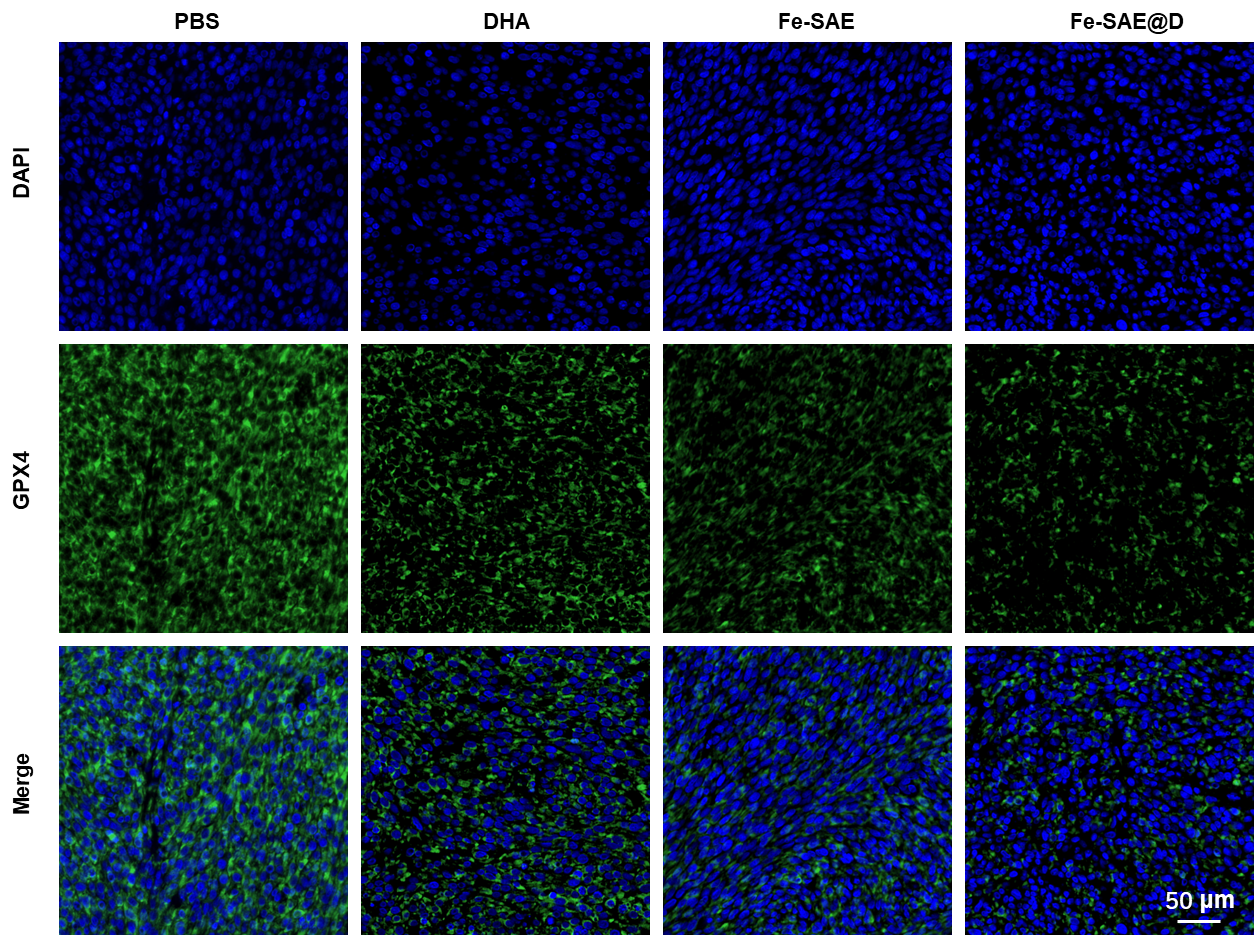


**Figure S36.** GPX4 staining of tumor tissue.
